# Probabilistic Critical Controllability Analysis of Protein Interaction Networks Integrating Normal Brain Ageing Gene Expression Profiles

**DOI:** 10.3390/ijms22189891

**Published:** 2021-09-13

**Authors:** Eimi Yamaguchi, Tatsuya Akutsu, Jose C. Nacher

**Affiliations:** 1 Department of Information Science, Faculty of Science, Toho University, Funabashi 274-8510, Japan; 6519009y@st.toho-u.jp; 2 Bioinformatics Center, Institute for Chemical Research, Kyoto University, Uji 611-0011, Japan; takutsu@kuicr.kyoto-u.ac.jp

**Keywords:** critical controllability, probabilistic controllability, brain, ageing process, protein networks, gene expression

## Abstract

Recently, network controllability studies have proposed several frameworks for the control of large complex biological networks using a small number of life molecules. However, age-related changes in the brain have not been investigated from a controllability perspective. In this study, we compiled the gene expression profiles of four normal brain regions from individuals aged 20–99 years and generated dynamic probabilistic protein networks across their lifespan. We developed a new algorithm that efficiently identified critical proteins in probabilistic complex networks, in the context of a minimum dominating set controllability model. The results showed that the identified critical proteins were significantly enriched with well-known ageing genes collected from the GenAge database. In particular, the enrichment observed in replicative and premature senescence biological processes with critical proteins for male samples in the hippocampal region led to the identification of possible new ageing gene candidates.

## 1. Introduction

Ageing is the direct consequence of multiple types of cellular and molecular deterioration and damage across the lifespan [1,2]. Among these, ageing is responsible for modifications in brain cognitive abilities, such as memory and reasoning, as well as changes in executive functions, including planning and abstract thinking. Most of these functions are orchestrated in the hippocampus (HC), which is located in the temporal lobe of the brain [3,4]. Although extensive studies based on gene expression changes have been performed in order to demonstrate the cognitive deterioration of patients with Alzheimer’s disease (AD) [5], there are far fewer large-scale computational analyses and models of transcriptomic changes observed in several healthy brain regions across the lifespan.

This gap was partially addressed by Berthold et al., who examined gene expression changes in the course of normal brain ageing [3]. Their analysis focused on selected brain regions. It is believed that the ability to form long-term memories from novel events depends on information processing within the temporal cortical regions (HC and entorhinal cortex (EC)). These are the main regions disrupted in AD and other mild forms of cognitive decline [3]. However, different brain regions may well undergo gene expression changes, even though these regions are more robust and potentially free from pathology ageing and, in particular, AD [5,6]. This is the case in the postcentral gyrus (PCG), located in the parietal lobe of the brain, which is responsible for the sense of touch. Berthold et al.’s analysis also included the superior frontal gyrus (SG), which is involved in self-awareness and is linked to the sensory system. While it was expected that most gene expression changes would be observed in the cognitive regions susceptible to age-related diseases, such as HC and EC, their results showed that the SG and PCG regions exhibited more extensive transcriptomic changes across the lifespan. Specifically, the SG showed the largest changes in elderly males [3,5].

Although this study was very important, more elaborate models of dynamic changes in ageing are necessary to investigate the changes in the normal brain across the lifespan. In particular, the integration of dynamic protein network information with gene expression data could aid in unveiling genes responsible for the ageing process. Moreover, this network can also be examined from a controllability perspective [7,8] in order to identify unique critical control genes responding to ageing.

**Figure 1:**
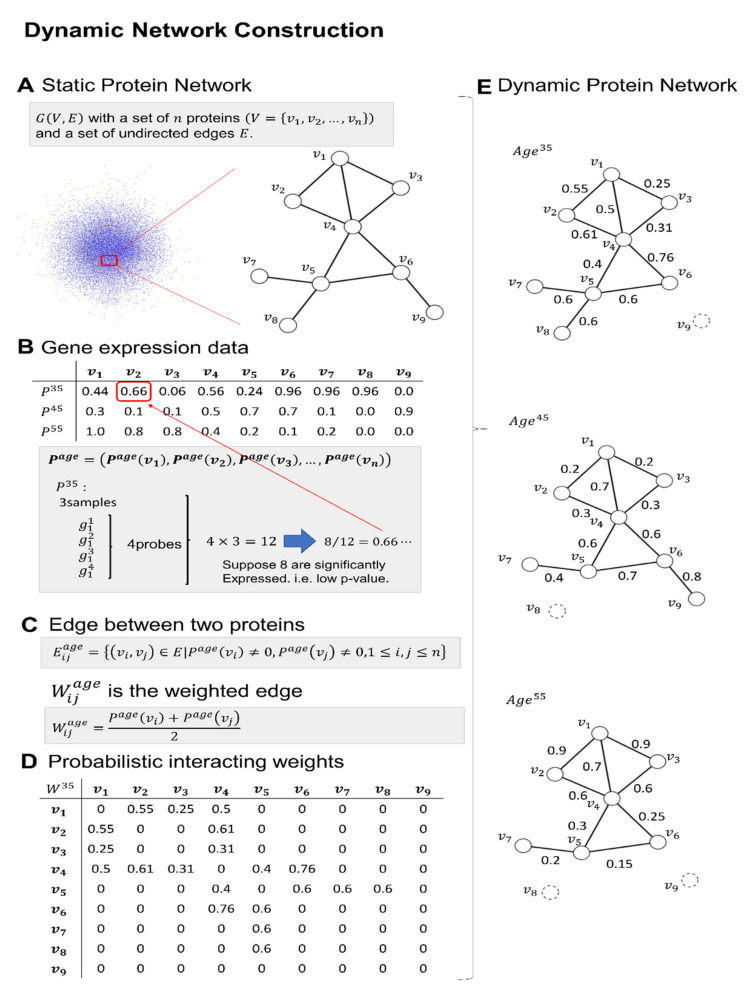
Illustration of the construction of dynamic weighted protein networks. (**A**) Example of a static protein network. The static protein network was constructed from the HINT database. (**B**) By using gene expression data, significantly expressed proteins were determined. (**C**) Edges were allowed only between expressed proteins at each age period. (**D**) The probabilistic weights were constructed using an arithmetic mean of each protein pair expression level. (**E**) Examples of dynamic protein networks at different age periods.

In this study, we collected the gene expression profiles of several normal brain regions, such as HC, SG, PCG and EC, from individuals aged 20–99 years. The gene expression profiles were subdivided into four age categories across 20-year increments, which were further integrated with human protein–protein interaction networks. Based on the significant expression changes in each age shift, we then generated a dynamic weighted protein–protein network for each different age range and each brain region. The interacting weight was considered an interaction probability (see Figure 1).

Recently, controllability approaches have been proposed for the analysis of complex biological networks and the identification of a minimum set of nodes that can control the entire network. Among them, maximum matching (MM) [7,9,10] and minimum dominating set (MDS)-based methods have been proposed [8,11]. In particular, the MDS method has been applied to many biological systems [11] and was recently used to uncover the control architecture of brain networks [12]. Several studies have shown that MDS is enriched in important biological functions and disease genes [11,13,14,15,16,17]. Because the MDS solution is not unique, we needed to classify the nodes into several control categories, namely, critical, intermittent and redundant nodes [18,19].

Efficient algorithms that identify these categories in directed and undirected networks in the context of the MDS controllability problem have already been proposed [20,21]. However, the large-scale dynamic protein network we constructed was much more complex because the edges were weighted and probabilistic, a problem that has not been solved before using an MDS approach.

**Figure 2:**
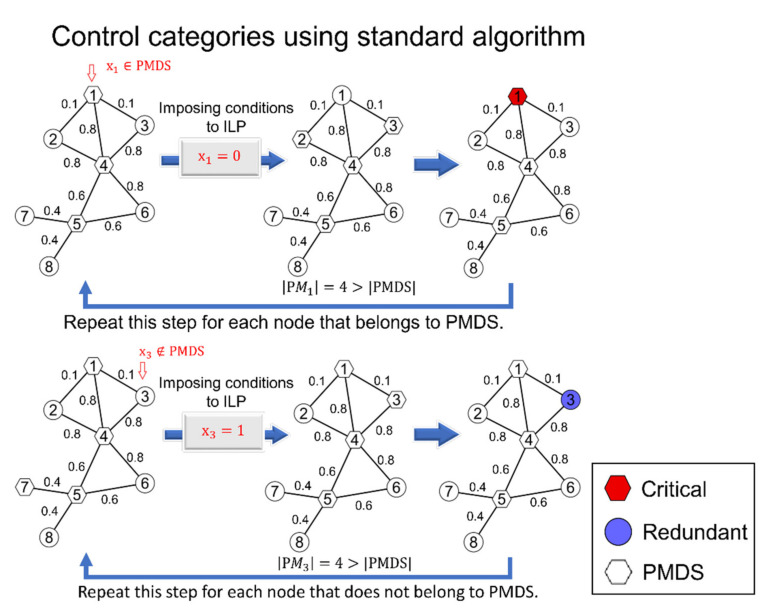
Illustration of the standard algorithm for determining control categories after preprocessing steps were performed on a probabilistic network. Note that each edge has a failure probability score. The preprocessing steps consisted of three mathematical propositions. See the Methods section for the complete proposed CPMDS algorithm.

**Figure 3:**
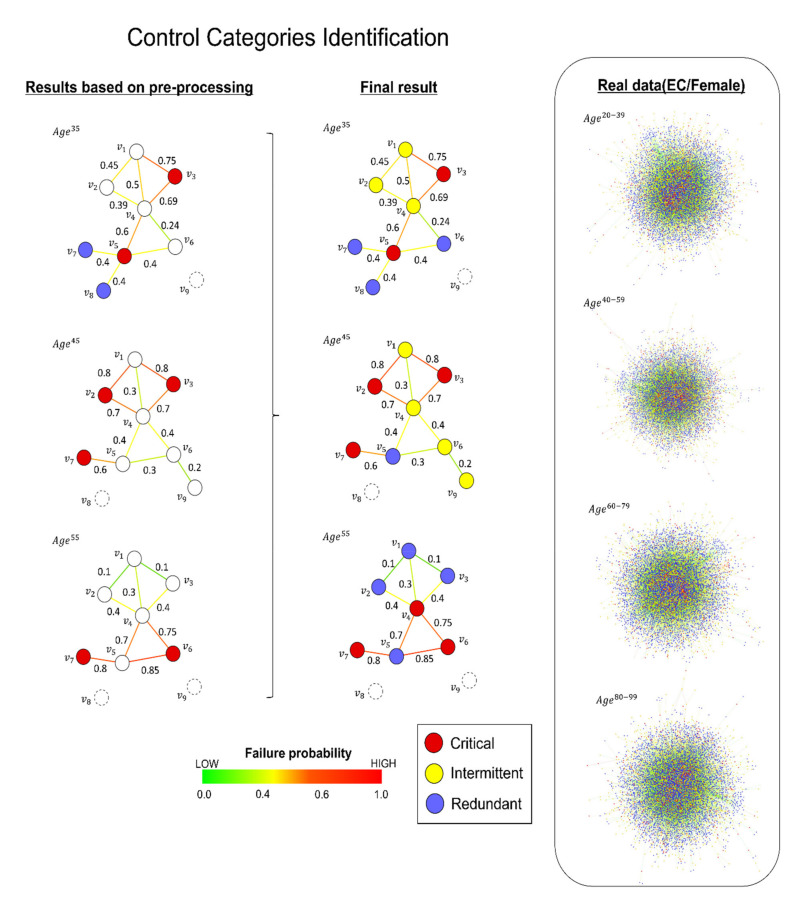
(**Left**) The network results for each control category after applying the preprocessing steps (see Methods section). Note that these results were obtained only by using mathematical propositions 1, 2 and 3, and no ILP computation was needed. (**Right**) The final result of each network after using ILP and the standard control categories algorithm, as shown in Figure 2. Each network edge had a failure probability at a given age that was calculated by subtracting to 1 the interacting strength of each interacting pair (see Equation (3) in Methods).

To address this computational problem, we proposed an efficient algorithm that identified critical, redundant and intermittent proteins (nodes) in the constructed probabilistic networks in the context of an MDS controllability framework. Critical nodes denote the set of nodes that appear in all the solutions of an MDS. An intermittent set refers to the nodes that appear in some, but not all, of the MDS solutions. Redundant nodes denote the nodes that are absent from all the MDS solutions. These three control categories could also be applied to classify the nodes in a more complex version of an MDS problem, such as the probabilistic minimum dominating set (PMDS) (see Methods for details). In this study, we focused on providing a new algorithm to efficiently identify critical control categories in a PMDS. The proposed critical probabilistic minimum dominating set (CPMDS) algorithm relied on several mathematical propositions that allowed us to greatly simplify the weighted network using a preprocessing computation, which significantly sped up the computation (see Methods section and Figure 2 and Figure 3). The remaining network was then solved using integer linear programming. By using this algorithm, we identified critical controllers that were significantly enriched by well-known ageing genes collected from the GenAge database across all the 20-year increments and brain regions.

The objectives and main findings of our analysis are summarized here:

(1) We developed a controllability algorithm based on MDS that efficiently identified critical nodes in large-scale probabilistic protein networks. Because of the weighted edges, it was a challenging problem to efficiently compute critical nodes among the multiple possible solutions. We bypassed this problem by introducing three novel mathematical propositions that significantly reduced the computational complexity and time, and extended the computable network size of the networks. This algorithm is one of the main theoretical contributions of this work.
(2) We validated the algorithm performance using artificially constructed weighted scale-free networks.
(3) To examine changes across time, the networks should not be a single snapshot, a time, or a convolution of different time steps. By using gene expression data, we constructed dynamic protein networks that were also weighted, which is probabilistic.
(4) By following steps (1) and (3), we examined whether the CPMDS network-based model identified critical proteins that were also associated with known ageing genes. The findings show that the identified critical controllers were significantly enriched by well-known ageing genes collected from the GenAge database, which was one of the main results of the data analysis.
(5) Critical ageing genes are also proteins enriched in many gene ontology (GO) annotations, showing the biological importance of these proteins. In particular, the enrichment observed in the replicative and premature senescence biological processes with critical proteins for male samples in HC brain regions led to the identification of possible new ageing-gene candidates.

## 2. Results

### 2.1. Computational Results from Artificial Scale-Free Networks

**Figure 4:**
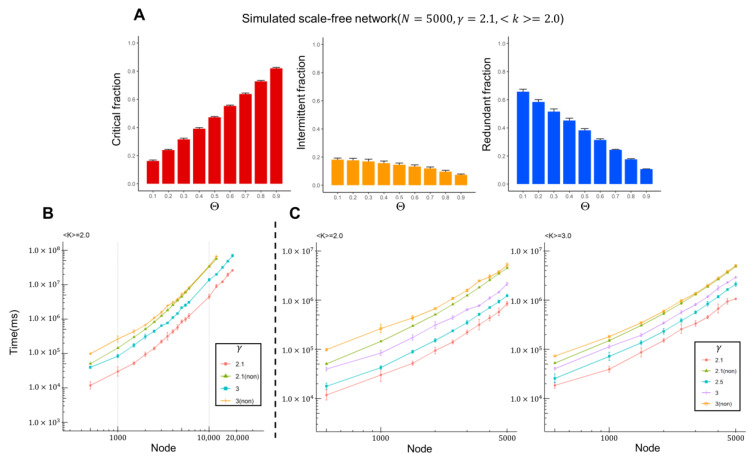
Computational results from artificial scale-free networks. (**A**) Control categories for each probabilistic threshold parameter Θ of CPMDS model. (**B**) Computational time vs. network size for scale-free networks with different degree exponents and average degree *<k>* = 2.0. Green (triangles) indicates the results of the algorithm without the preprocessing step. (**C**) The computational time effect of increasing the average degree from <*k*> = 2 (**left**) to <*k*> = 3 (**right**).

Before the discussion of our analysis of real brain data, we will demonstrate the efficiency of our proposed algorithm using simulation data. We constructed undirected scale-free networks with 5000 nodes, an average degree *<k> =* 2, and a degree exponent γ = 2.1. We then assigned random values [0, 1] to each edge to construct a weighted network. To construct these networks, we used the Havel-Hakimi algorithm with a random Monte Carlo edge swaps (HMC) model [22]. We then applied the proposed algorithm (see Methods section) and computed the control categories at different probability thresholds Θ from 0.1 to 0.9. The results suggest that the critical, redundant and intermittent fractions of nodes showed a different tendency as the probability threshold increased (see Figure 4A). In general, it is easier to control a network when the probability threshold is lower, which leads to a smaller fraction of critical controllers. As described in the Methods section, the algorithm used three newly proposed propositions that significantly reduced the computational time. These propositions preprocessed the network structure and classified a large number of nodes into the control category, simplifying the subsequent ILP computation. We constructed scale-free networks with up to 18,000 nodes and calculated the computational time from the newly proposed algorithm, as well as from an algorithm without those propositions, which we also proposed in this study. The results show that the method with the newly proposed propositions speeds up the computational time more than sevenfold (see Figure 4B). The algorithm also expanded the network size, since it could compute up to 18,000 nodes. The algorithm without preprocessing steps could only solve networks of up to 12,000 nodes (see green line with triangles) within a feasible time. Note that the computational time increased exponentially, as shown in SI Figure S1. We also investigated the effect of network parameters, such as the degree exponent and average degree. As the degree exponent γdecreased, the efficiency of the algorithm increased (see Figure 4B,C and Figure S2). The scale-free networks with lower degree exponents tended to have hubs with higher degree nodes, making them easier to control. On the other hand, when the average degree increased, the computational time tended to increase slightly (see Figure 4C and Figure S2), which was consistent with an increment of complexity by adding more edges to the network. For each experiment, a set of five networks was constructed, and the Figures show the average results.

### 2.2. Critical Control Proteins Are Significantly Enriched and Associated with Ageing Genes across the Lifespan

**Figure 5:**
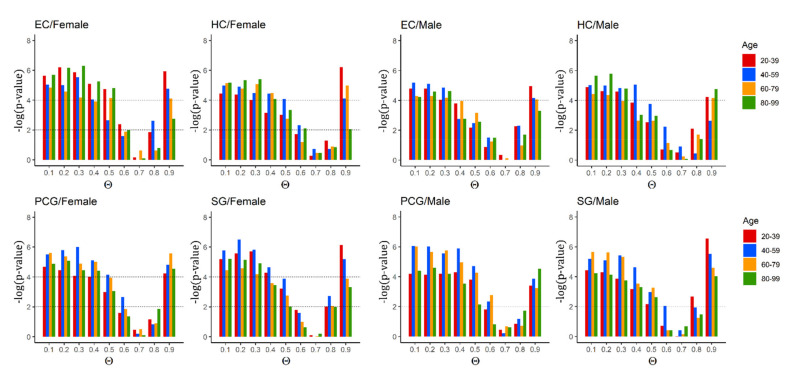
Critical control proteins were significantly enriched and associated with ageing genes across the lifespan from Θ = 0.1 until Θ = 0.5, where Θ = 0.6 shows a transition point. From Θ = 0.7, the increasing bars denote the increasing statistical significance of the depletion of critical protein with known ageing genes. The results are shown for all four brain regions, for each age period and for females (**left**) and males (**right**). Note that *p*-values are shown as the −log(*p*-value) in the vertical axis. The horizontal axis indicates the probabilistic parameter Θ.

Our proposed CPMDS method allowed us to associate critical, redundant and intermittent control roles to each protein. First, we focused on whether the critical category was sufficiently enriched with well-known ageing genes. Figure 5 illustrates the influence of the parameter Θ in the model. Specifically, it tells us how the statistical significance of the critical proteins associated with the well-known ageing genes changed when the Θ parameter increased. The results indicate that the critical set was significantly associated with ageing genes for most Θ probabilities, especially for Θ < 0.6 (*p*-value <0.001) (see Figure 5). The *p*-values were calculated using a two-tailed Fisher’s exact test. This finding was maintained across the entire lifespan, which was divided into four 20-year increments. The results were assessed independently for males and females, in each brain region.

**Figure 6:**
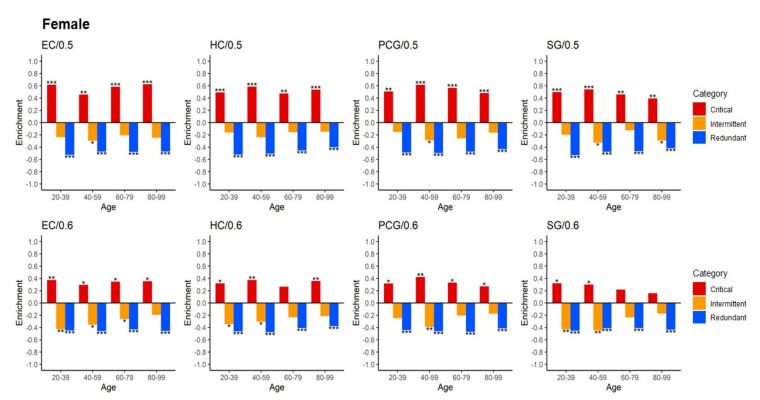
Enrichment values for the association of proteins with well-known ageing genes vs. each age period and each control category. Female samples for all four brain regions at three values of threshold probability Θ. See Supplementary Information file for the male samples. The following symbols *** denote *p*-value < 0.001, ** (*p*-value < 0.01), and * (*p*-value < 0.05). Negative enrichment indicates depletion.

In particular, there was a transition point in the enrichment towards depletion located precisely at Θ = 0.6. As Figure 5 shows, for many tissues and age ranges, Θ = 0.6 still achieved a statistically significant enrichment, but for some tissues and age ranges, this enrichment decreased more and was not significant. From Θ = 0.7, the enrichment changed into depletion, and this depletion of critical proteins with known ageing genes became more statistically significant when the Θ value increased to 0.8 and 0.9. Note that the bar indicates −log(*p*-value). In other words, until approximately 0.6, the *p*-value was very small (high bars because −log(*p*-value)), which indicated a significant association between critical and ageing genes. There was no significant association at Θ = 0.7, so the null hypothesis (no association/correlation between critical/noncritical and ageing/nonaging) was satisfied. When Θ increased more, the enrichment was negative (see also Figure 6 and Figures S3–S6 in SI) and the *p*-value decreased again (high bars because log(*p*-value)) and deviated from the null hypothesis again; this time, however, this indicated a significant association between critical and non-ageing genes (that is, a depletion in the association between critical and known ageing genes).

Next, we investigated how the enrichment level of each control category changed during ageing. The results are shown in Figure 6 and Figure S6 in SI for females and males, respectively. The statistical significance of the enrichment values is shown using the following symbols: *** denotes *p*-value < 0.001, ** denotes (*p*-value < 0.01), and * denotes (*p*-value < 0.05). Negative enrichment indicates depletion. The *p*-values were calculated using a two-tailed Fisher’s exact test. There was a significant enrichment of critical proteins with ageing genes (red) and a strong depletion of intermittent (blue) and redundant (yellow) proteins for Θ = 0.5 (see also Figure S3 in SI with results for Θ < 0.5 with the same tendency). This means that the CPMD model identified critical proteins that are significantly enriched with known ageing genes, which was one of our main results.

**Figure 7:**
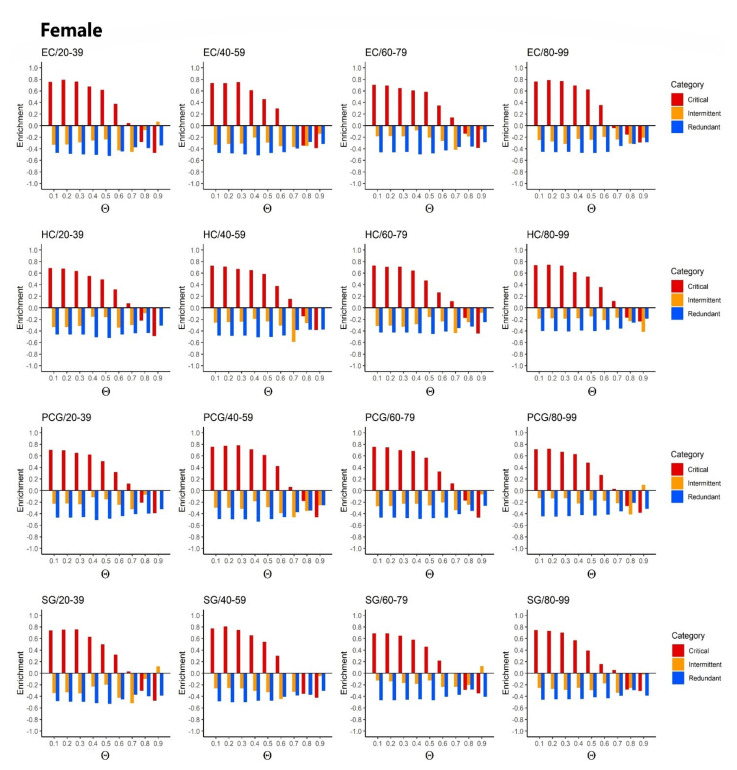
Enrichment values for the association of proteins with well-known ageing genes vs. each threshold probability Θ. The figure shows the results for female samples and all four brain regions and all age periods. The associated *p*-values, calculated using a two-tailed Fisher’s exact test, are shown in Table S2 (Excel file).

As discussed in Figure 5, the case of Θ = 0.6 revealed a transition point for the enrichment. This is shown in Figure 6, in which the female SG displayed a small, nonsignificant enrichment among the larger age ranges, 60–79 and 80–99. This decrease in enrichment seemed even more severe in males. While the PCG region still showed enrichment in females in the 60–79 and 80–99 age ranges, in males, the enrichment was not significant for the 80–99 age range (see Figure S6 in SI). For HC in females, only 60–79 showed no significant enrichment, whereas in males, except for the 40–59 age range, there was no significant enrichment. For the female EC region, all age ranges showed significant enrichment, while only the 40–59 and 80–99 age ranges showed significant enrichment in males. Therefore, we believe that the value Θ = 0.6 is useful for assisting in the observation of differences between female and male samples. See also Figure 7 and Figure S7 for an overview of this transition for all tissues and parameter values for females and males, respectively.

The observed stability of EC and HC regions was in agreement with Berthold et al. [3], whereas PCG and SG seemed to be more sensitive to ageing. In particular, it was observed that changes in the largest number of differentially expressed genes between ageing groups were related to the activity of the cortical regions of the brain, in particular the SG and PCG. By contrast, the HC and EC regions showed the fewest genes responsive to ageing. Although their study and our analysis are technically very different, and it is difficult to perform a quantitative comparison because we added a protein interaction network layer and performed a controllability analysis rather than a direct computation of gene expression changes, the observed activity in the enrichment changes for SG and PCG after the sixth and seventh decades shown in Figure 6 suggests a qualitative agreement with Berthold et al.

The complete results with all probability thresholds Θ from 0.1 to 0.9 for females and males, respectively, are shown in SI Figures S3–S6. In general, when increasing the probability threshold Θ, it became more difficult to control the network. Therefore, the size of the critical set became larger, which decreased the enrichment of ageing genes. Indeed, for probabilities of Θ≥0.7, there was almost a statistically significant full depletion in all age categories (see Figure 7 and Figures S3–S7 in SI.)

### 2.3. Identified Critical Ageing Proteins Are Dynamically Assigned across Ageing

**Figure 8:**
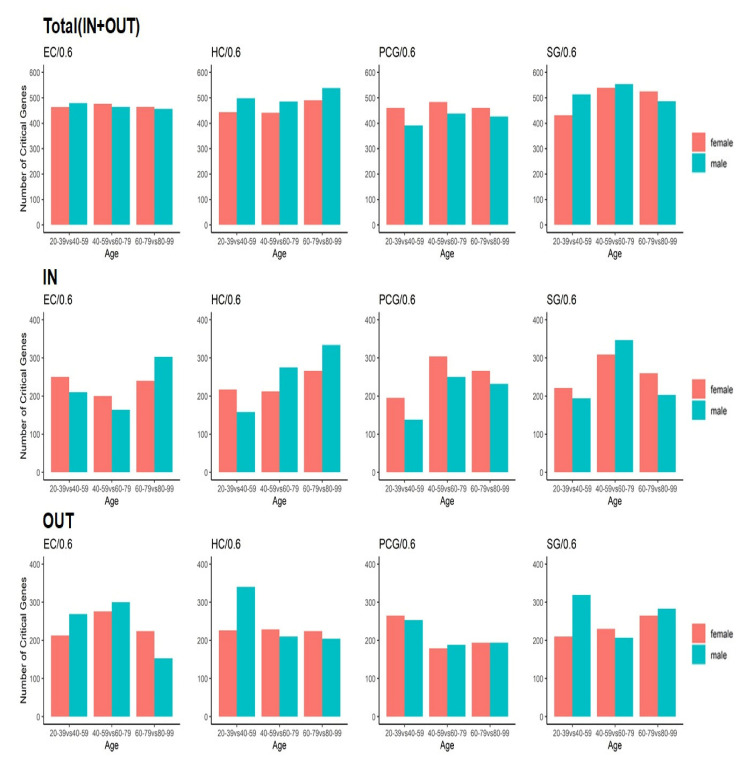
Distribution of the number of critical proteins that switched the control category between two consecutive age periods (horizontal axis) and computed for each threshold probability Θ = 0.6. The results for all four brain regions in females and males are shown. The total sum of the number of newly added critical proteins (IN) and the number of critical proteins that switched to different categories (OUT) is shown in the top row.

The analysis presented above shows how the number of critical nodes changed across the lifespan by increasing or decreasing their statistical enrichment with ageing genes. However, it does not tell us precisely whether the same proteins engaged critical control roles with ageing across the lifespan. It may be that the number of critical proteins did not change (some examples suggested this, as shown in Figure 5), but the individual proteins that actually played a critical role were replaced in large numbers by others across the lifespan. To shed light on this issue, we computed the number of critical proteins that changed role over each 20-year increment. For example, if there was a critical set of proteins composed of *p1*, *p2*, *p3* in the age category of 20–39 years and the set changed into *p1*, *p2*, or *p4* in the age category of 40–59 years, we considered that the number of changes in the critical set was two, even though the size of the critical set was still three. We were able to further specify the origin of the changes by considering the number of newly added critical proteins (INs) and the number of critical proteins that switched to different categories (OUTs). In the example, there was one IN-protein *p4* and one OUT-protein *p3*. The results are shown in Figure 8 and suggest that there was a tendency to increase the replacement and addition of new proteins in the critical control category in the transition for age ranges computed at Θ = 0.6. For example, in females, for the two consecutive transitions from 40–59 vs. 80–99 years for SG and 60–79 vs. 80–99 years for SG, the number of changes in critical nodes reached the highest point. In males, EC showed a decreasing tendency, whereas HC increased with age, with a peak at 60–79 vs. 80–99 years. PCG and SG also showed peaks at 40–59 vs. 60–79 years.

According to Berthold et al., most gene changes in terms of gene expression occur in the age range of 40–59 vs. 60–79 for males, especially SG, followed by PCG and HC, and increasingly change for females across the lifespan. The observed tendency towards increasing changes in critical proteins in the HC and SG regions for aged cohorts in females and the peaks observed for PCG and SG in middle-aged transitions in males are in qualitative agreement. Again, we also suggest that there are differences in tendencies in both analyses because in this study we identified changes in critical controllers using a dynamic network as the background, instead of measuring only gene expression profile change.

To visualize the changes in all the critical proteins across the different age categories and different brain regions, we also constructed heat maps (Figure S13). These demonstrated the changes not only in consecutive age transitions, as shown in Figure 8 and Figure S12, but also across all lifespan combinations (see Figure S13).

The number of critical, intermittent and redundant genes that changed the control category across the lifespan can be visualized in Figures S8–S11 for each brain region. The thickness of the grey lines is proportional to the number of proteins. For all brain regions, some critical nodes tended to become intermittent or redundant and vice versa, which shows the dynamic nature of the ageing process. The transition was even stronger between redundant and intermittent nodes.

### 2.4. Unique Critical Control Proteins across Lifespans

Closely related to the analysis shown in Figures S8–S11, here, we shall focus on our analysis of the genes that were uniquely assigned to critical control roles at specific ages and brain regions. This means proteins that were identified as critical proteins only during a specific age range. This allowed us to analyse from a different perspective and provided complementary insights to those shown in Figure 6, Figure 7 and Figure 8. Here, we shall focus on Θ = 0.5 rather than the transition point because it showed significant enrichment across the lifespan, as shown in Figure 6. The Venn diagram shown in Figure S14 offers an example for male HC tissue at Θ = 0.5 of the numbers of critical nodes unique to an age period and shared across ages. The results for all brain regions and the probabilistic threshold values are shown in Figures S15–S18 for female samples and Figures S19–S22 for male samples.

Next, we computed the fraction of unique critical proteins at specific ages and brain regions for male samples (see Figure S23). For example, at Θ = 0.5, the fraction of critical proteins for HC showed the most robust tendency towards increase across the lifespan, duplicating its initial number at the age range of 80–99. Note that, as demonstrated in Figure 8, the male HC also showed an increasing tendency towards IN-critical proteins with age. This indicated that the underlying protein network in the HC region of the brain underwent the largest changes, and that these changes rewired the network in such a way that they make the network more difficult to control, requiring an increasing number of unique critical proteins. This correlates with previous reports indicating that the HC region suffers more deterioration with ageing. PCG showed a similarly increasing pattern (see the positive correlation between PCG and HC regions (red) in Figure S24). SG, especially EC, showed a decrease in critical node faction with age and showed a positively correlated pattern between themselves. Interestingly, the HC-PCG vs. EC-SG pairs were negatively correlated (Figure S24).

For female samples, we observed that the critical fraction showed an overall increasing tendency for all tissues across ageing, although the variations across ages were significantly smaller (most correlations were positive (red) for Θ≤0.5) (see Figures S25 and S26). This suggests that the female brain tissues actually suffered a smaller number of changes in unique critical proteins than those of males across ages. Note that the fraction of PMDS (see methods for details) was approximately 20% (see Figures S27 and S28), and that the fraction of critical proteins (see Figures S29 and S30) was approximately 10% and roughly remained constant across the lifespan for both males and females. Therefore, the strength of the fluctuations observed in unique critical proteins for specific ages (Figures S23–S26) are relevant.

### 2.5. Ageing Proteins Identified as Critical Controllers Are Enriched in Gene Ontology Functional Categories

**Figure 9:**
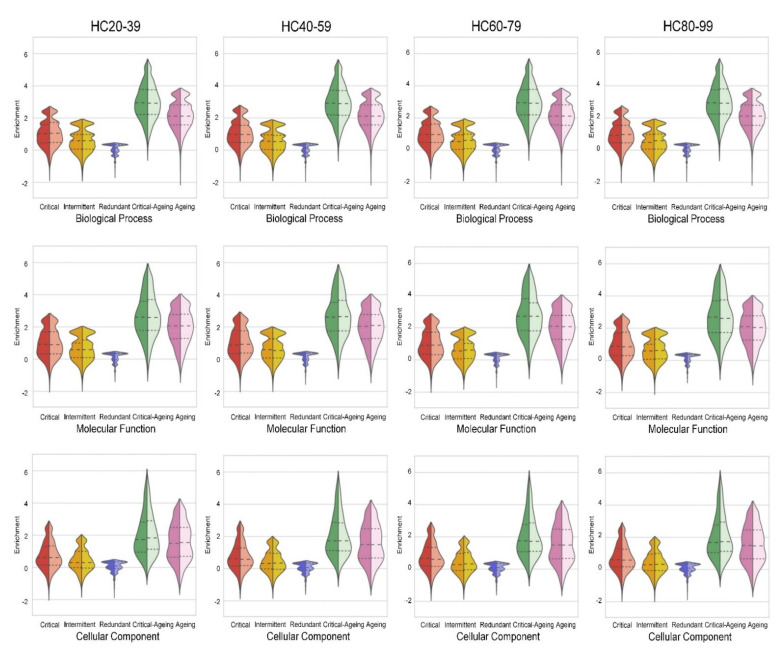
Violin-style plot of the proteins enriched for each gene ontology annotation in the HC brain region and each age period computed at probability Θ=0.5: biological process, molecular function and cellular component. From left to right: enrichment of critical, intermittent and redundant proteins with each GO annotation. Ageing proteins identified as critical controllers showed the largest enrichment in the gene ontology functional categories. The last plot indicates the enrichment of well-known ageing genes in each GO annotation category. Each split violin plot shows the probability density of data in its left half (male) and right half (female) for comparison. The median for males and females was very similar and showed the largest enrichment for the critical-ageing category in both genders.

To examine the biological functions of the uncovered critical controllers and to compare them with those of ageing proteins, we performed a gene ontology analysis using information on biological processes, molecular processes and cellular components from the UnitProt database. Each protein was then classified according not only to its control category (critical, redundant or intermittent), but also according to whether it encoded a well-known ageing gene or whether it encoded a well-known ageing gene and is engaged in critical control (critical ageing category). Next, we computed the enrichment for each category, and the results showed that the critical ageing category exhibited the largest enrichment in all the gene ontology categories (see the violin-style plot in Figure 9 for HC tissue and Figures S31–S33 in SI for the rest of the brain tissues). This tendency was shared by all four analysed brain regions. Moreover, female and male samples were analysed independently. Each split violin plot showed the probability density of data in its left half (male) and right half (female) for comparison. Because we included all the GO annotations, the changes between males and females in the probability density of data in each plot were not significant, even though there were very small changes in the median and quartile values. To further analyse the biological functions associated with the critical control, we examined each annotated term and functional class inside each GO category. The compiled results are available as supplementary files (see the Excel files Tables S7–S18 for all GO categories). The most significantly enriched GO terms (biological process) with critical proteins were largely shared by several brain regions and ages (Tables S3–S6). For example, the Fc-gamma receptor signalling pathway involved in phagocytosis (GO:0038096) was significantly enriched in EC20–39 (*p* = 1.12 × 10^−6^; all the *p*-values were derived from two-tailed Fisher exact tests), HC20–39 (5.37× 10^−8^), PCG60–79 (*p* = 1.17× 10^−6^), and SG80–99 (*p* = 1.37× 10^−6^) in males. The histone mRNA catabolic process (GO:0071044) was significantly enriched in multiple regions and ages, such as EC40–59 (*p* = 4.48× 10^−6^) and EC80–99 (*p* = 3.30× 10^−6^) in females, SG40–59 (*p* = 4.51× 10^−6^) and SG80–99 (*p* = 5.68× 10^−6^) in females, HC40–59 (*p* = 2.42× 10^−6^) and HC60–79 (*p* = 3.55× 10^−6^) in females, and PCG20–39 (*p* = 3.36× 10^−6^) and PCG40–59 (*p* = 2.88× 10^−6^) in males. Finally, the transforming growth factor beta receptor signalling pathway (GO:0007179) appeared to be significantly enriched in EC60–79 (*p* = 8.64× 10^−7^), SG20–39 (*p* = 2.88× 10^−6^), SG60–79 (*p* = 2.70× 10^−6^), and HC20–39 (*p* = 3.48× 10^−6^) in females and in HC60–79 (*p* = 6.17× 10^−7^) and PCG80–99 (*p* = 9.04× 10^−6^) in males. This also suggested differences in the ranking of enrichment for GO annotations in males and females. As shown in Tables S3–S18, the ranking order of functional categories changed across the lifespan. This shows that our method allowed us to obtain precise insights into the biological functions related to both the ageing process and critical controllability.

**Figure 10:**
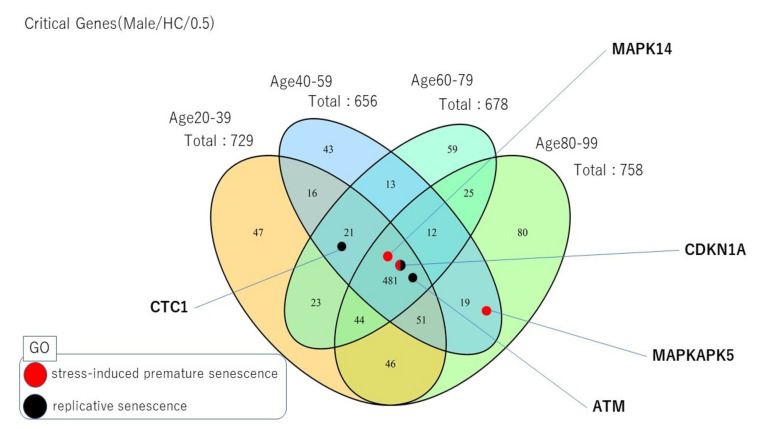
Venn diagram for male HC brain tissue at Θ = 0.5 showing the numbers of critical proteins for each age period and those shared across ages. The possible critical ageing protein candidates that were identified were related to stress-induced premature senescence and replicative senescence.

We further investigated GO terms (biological process) related to ageing, such as stress-induced premature senescence and replicative senescence, for the HC brain region in males at Θ = 0.5 (see Figure 10), whose fraction of unique critical proteins showed the most robust tendency towards increase across the lifespan (Figure S23). Although the results shown in Figure 8 and Figure S23 are complementary, the computations performed in Figure 8 show changes in the number of critical proteins across the age range, whereas the results shown in Figure S23 indicate the fraction of unique critical proteins at specific ages. Note, however, that the number of changes in critical proteins for HC steadily increased with the age ranges for Θ = 0.5 and reached a peak at 60–79 vs. 80–99 years for Θ = 0.6 (see Figure 8 and Figure S12). In general, cellular senescence refers to the process in which cell division is suppressed to protect against cancer. Moreover, its relation with age-related processes and telomere attrition, among other processes has been suggested [23,24,25]. The stress-induced premature senescence process was enriched with critical proteins (see Figure 10 and Table S1 in SI) in the male HC brain regions across ages. Specifically, this GO term was enriched with MAPK14, CDKN1A, and MAPKAPK5 genes that encoded critical proteins at HC40–59 (*p* = 6.14× 10^−4^, E = 2.46; all the *p*-values were derived from two-tailed Fisher exact tests, and *E* denotes the enrichment level) and HC80–99 (*p* = 8.01× 10^−4^, E = 2.37). MAPK14 and CDKN1A genes are well-known ageing genes, but MAPKAPK5 was not previously reported as an ageing gene, according to the GenAge database. The replicative senescence process was also significantly enriched with critical proteins for all age ranges except the last one: HC20–39 (*p* = 0.01, E = 1.68), HC40–59 (*p* = 0.01, E = 1.77), HC60–79 (*p* = 0.01, E = 1.72), and HC80–99 (*p* = 0.13, E = 1.12). In particular, our analysis shows that the ATM and CDKN1A genes that encoded critical proteins across all age periods were associated with replicative senescence biological processes. CTC1 was also identified as a critical control protein across all age ranges except for the last one and played a role in the replicative senescence process (see Figure 10 and Table S1). Interestingly, ATM and CDKN1A are well-known ageing genes according to the GenAge database. By contrast, CTC1 is not listed as an ageing gene in the database. However, recent reports suggest that it is closely related to the ageing process [26,27]. The complex CST, which consists of CTC1, STN1 and TEN1 proteins, plays a key role in telomeric DNA replication processes [27]. In fact, telomere shortening across the lifespan has been suggested as a method for predicting biological ageing [28,29,30]. For the rest of the brain regions, we refer to Tables S3–S18.

## 3. Discussion

While previous studies have focused on transcriptome changes in the normal brain, age-related changes in the brain have not been investigated from a network controllability perspective. Our analysis went beyond the straight analysis of gene expression levels and integrated the underlying protein network within a controllability framework. This approach allowed us to capture dynamic changes of the network in ageing by proposing a new critical probabilistic controllability model that identified critical proteins for each age period.

The data analysis revealed that the identified critical proteins were significantly enriched with well-known ageing genes collected from the GenAge database across all 20-year increments and brain regions.

Specific and different abundances of unique critical proteins were identified at each age period, which suggests that network changes and controllability responses organize the brain regions in two specific groups, namely, EC-SG and HC-PCG, for males. The regions in each group showed positively correlated behaviour. However, the groups showed opposite responses to age, leading to negative correlations between them. By contrast, female samples mostly showed a tendency towards a globally positive correlation with ageing for all brain regions.

In particular, the enrichment observed in the replicative and stress-induced premature senescence biological processes with critical proteins in the HC brain region in males led to the identification of possible new ageing gene candidates. It is well known that during senescence, cells cannot divide indefinitely in an attempt to prevent or curtail tumorigenesis. However, several works have also suggested a relationship between the senescence process and the ageing process, ageing-associated pathologies and telomere attrition, among others [23,24,25]. The data analyses determined that the stress-induced premature senescence process was enriched by critical proteins in the HC male region. Namely, the MAPKAPK5 gene encoded a critical protein at several age periods, together with the MAPK14 and CDKN1A genes. While the MAPK14 and CDKN1A genes are considered well-known ageing genes, the MAPKAPK5 gene is not yet included in the GenAge database. Therefore, our study suggests that it as an ageing gene candidate. MAPKAPK5 encodes a protein that belongs to the serine/threonine kinese family and plays a role as a tumour suppressor [31]. Interestingly, this gene has been associated with several diseases, especially one type of aggressive brain tumour, the glioblastoma mesenchymal subtype [31]. A similar analysis showed that the replicative senescence process was also enriched with critical proteins, such as ATM, CDNKN1A and CTC1. Among them, the CTC1 gene had not been reported previously as an ageing gene in the GeneAge database. Recent reports, however, suggested its role in the ageing process [26,27], as well as in telomeric DNA replication processes, via the protein complex CST [27]. Interestingly, the telomere shortening process was associated with the ageing of cells [28,29,30]. The CTC1 gene encodes a protein component of the protein complex CST. CST Telomere Replication Complex Component 1 is involved in telomere maintenance in order to prevent degradation. CTC1 gene mutations may lead to various diseases, such as cerebroretinal microangiopathy [31].

In summary, this study presented a novel computational method for identifying critical control proteins in complex probabilistic networks. The method used three newly derived mathematical propositions that allowed us to efficiently reduce the computational time significantly and solve the ILP problem in large networks. The method was applied to large-scale artificial scale-free networks in order to evaluate algorithmic performance. By integrating normal brain ageing gene expression profiles with protein networks, we constructed dynamic ageing networks for each brain tissue, which were systematically analysed by the proposed controllability method, identifying a specific set of critical proteins. These critical control proteins were significantly associated with ageing genes in each time period. These results suggest that our controllability method identified proteins that tended to be encoded by ageing genes. Additional data analysis, which was performed by combining gene ontology terms, revealed some ageing gene candidates.

Although we limited our study to a normal brain analysis, further integrated network controllability analyses can be performed to investigate not only normal brain tissues but also disease-affected brains (e.g., Alzheimer’s disease) [5], as well as different types of tissues, such as skin [32]. Specifically, in addition to the brain areas responsible for cognitive function and memory, the regions responsible for exercise or motor neurons, such as the basal ganglia or the motor association cortex, should be analysed. Because the analysed gene expression profiles did not include tissue samples from these regions, the exploration of these brain areas was left for future study.

From a modelling viewpoint, analyses of critical probabilistic controllability using directed networks such as metabolic pathways and gene regulatory networks were also left for future study. Moreover, our analysis can be extended by integrating the protein interaction network with extracellular matrix interactions [ECM] [33], which is thought to play a central role in biological processes related to ageing.

## 4. Methods

### 4.1. Gene Expression Data at Different Ages

In this study, we used gene expression data from four different human brain regions: SG, PCG, EC and HC. The dataset consisted of 173 postmortem brain tissue samples from 55 individuals classified into four age ranges: 20–39, 40–59, 60–79 and 80–99 years [3]. Affymetrix HgU133plus2.0 microarray chips were used to profile gene expression and included 54,675 probes for each tissue sample. The dataset was downloaded from GEO database file series GSE11882 [3]. The probe IDs were mapped to protein/gene IDs using the UniProt database. This step was necessary to map gene expression data onto the underlying protein interaction network.

For each tissue sample, we computed the detection *p*-value from each expression data, which measured the statistical significance of the mRNA amount in the sample. We used an R-language (Bell Labs, USA) implementation of the MAS 5.0 software package [34]. The software executed the Wilcoxon signed rank-based gene expression presence/absence detection algorithm and classified the probes into three categories, P, M and A: present (expressed) (score 1), marginal (borderline) (we considered 1 or 0) and absent (not expressed) (score 0). The software used a statistically significant *p*-value < 0.04 as a threshold to determine the expressed (P) category of a probe. Small *p*-values implied the presence of transcripts, while large *p*-values implied the absence of transcripts [35]. Here, we used a majority vote technique to evaluate the expression level of a gene at a given age. This method was also used by Faisal and Milenkovic et al., in 2014, and Kawakami in 2017, to construct dynamic unweighted protein networks [36,37]. Because there were multiple samples per age and each gene may also have had several probes, we considered several contributions to the final expression level of a gene. Assuming that a gene *g* had *q* probes in a sample and that there were *y* samples at age *a*, then according to the majority rule the gene *g* would be expressed at a given age and if more than 50% of *q x y* probes, or at least (*(q x y)/2+1)*, were expressed. As shown in Figure 1B, gene *v_2_* had three samples at age 35, and there were four probes for each sample. Using MAS 5.0 software, out of 12 possible values, only 8 were expressed. Here, we differed from Faisal and Milenkovic, and instead of assigning a score value of *P^35^(v_2_)* = 1 for this gene (expressed), we assigned the fraction value *P^35^(v_2_) =* 8/12 = 0.66 because we aimed to construct dynamic weighted protein networks. Subsequently, we repeated this process for all the genes in the dataset.

### 4.2. Static Protein Interaction Network

A human protein interaction network was downloaded from the HINT (**H**igh-quality **INT**eractomes) database [38]. This database contains a curated compilation of high-quality protein–protein interactions from eight interactome resources, namely, BioGRID, MINT, iRefWeb, DIP, IntAct, HPRD, MIPS, and the PDB. The protein network is a graph that can be defined as *G(V,E)*, where *V = {v_1_, v_2_,…,v_n_}* is the set of nodes that represent proteins and *E* is the set of edges that indicate undirected interactions between protein pairs. The numbers of the nodes and edges are *N* = 10,791 and *E* = 47,427, respectively.

### 4.3. Ageing Genes Database

The proposed algorithm identified a set of controllers in four age categories and each brain region. We then used well-known ageing genes to determine whether the identified controllers, especially critical proteins, were significantly enriched by these ageing genes. The set of well-known ageing genes consisted of 307 genes that were collected from the GenAge database v19.0 [39].

### 4.4. Construction of the Dynamic Weighted Protein Interaction Network

To construct the dynamic weighted network, we integrated information from the static network and gene expression data as follows (see Figure 1C). If two proteins encoded by genes *v_i_* and *v_j_* at a given age were such that both *P^age^(v_i_)* and *P^age^(v_j_)* scores were nonzero and its edge *(v_i,_ v_j_)* also existed in the static network, then an edge was also at least present in the dynamic network at that given age:

(1) $$E_{ij}^{age}=\{(v_{i},v_{j})\in E|P^{age}(v_{i})\neq 0,\;P^{age}(v_{j})\neq 0,\;1\leq i,\;j\leq n\}$$

Because this network was still unweighted, we added a weight to each present edge. This was performed by averaging the score values *P^age^(v_i_)* and *P^age^(v_j_)* of each gene pair:

(2) $$w_{ij}^{age}=\frac{P^{age}(v_{i})+P^{age}(v_{j})}{2}$$

Hence, Equation (2) is the weight assigned to each present edge defined by Equation (1). As shown in Figure 1D, it led to a weighted score matrix that was used to construct dynamic weighted networks at a given age. Figure 1E shows several networks constructed at different ages. Note that not only did weighted scores change but also some edges were absent at some specific ages.

The above weighted analysis used an arithmetic mean metric. However, we were also able to explore different metrics, such as a geometric mean defined as $w_{ij}^{age}=\sqrt{P^{age}(v_{i})\cdot P^{age}(v_{j})}$.

Indeed, we recomputed our analysis using both metrics, and although some changes were observed, these were not large; moreover, they did not change the overall observed tendencies.

### 4.5. Standard Probabilistic Control Model

We first considered a probabilistic control model that has been successfully applied in different contexts [17,40]. In real-world complex networks, from biological to technological systems, the edges between nodes may represent protein interactions, metabolic reactions or electric power transmission lines. However, in all these molecular interactions or physical lines, probabilistic failures may occur. We were therefore able to define the probability of interacting failure between proteins $\left(v_{i}^{},v_{j}\right)$ ($\rho_{ij}^{age}$) at a given age and integrate it into a probabilistic control model. The failure probability at a given age was calculated by subtracting to 1 the interacting strength of each interacting pair at a given age as follows:

(3) $$\rho_{ij}^{age}=1-w_{ij}^{age}$$

The objective was to cover each protein with multiple nodes in the MDS so that the probability that at least one interacting edge would be active was at least Θ. Recall that the standard MDS is a minimum set of nodes *S* such that each node belongs to *S* or has a neighbour in *S*. For details of a standard MDS approach to controllability, see refs. [8,9,10,11]. The probabilistic extension can be formulated as a PMDS as follows. Let *S* be a dominating set. Next, we require *S* to satisfy:

(4) $$1-\prod_{v_{j}\in S\cap N(v_{i})}\rho_{ij}>\Theta \;\mathrm{or}\;v_{i}\in S,\;\mathrm{for}\;\mathrm{all}\;v_{i}\in V\;$$

which can be rewritten as:

(5) $$\sum_{v_{j}\in S}-\ln(\rho_{ij})\geq -\ln(1-\Theta )$$

for $v_{i}^{}\notin S,\;$where $\rho_{ij}^{}$ indicates the probability of failure between proteins $\left(v_{i}^{},v_{j}\right)$.

The standard MDS problem was formalized by the following integer linear programming (ILP) problem:

*Minimize* $\sum_{v_{i}\in \;V}x_{i}$

subject to:

(6) $$x_{i}+\sum_{(v_{i},v_{j})\;\in \;E}x_{j}\geq 1$$

(7) $$x_{i}\in \{0,1\},\;\forall \;v_{i}\in V$$

where an MDS is obtained by the set $\left\{x|x_{i}=1\right\}$, *V* denotes the set of protein nodes in the protein network and *E* indicates the set of undirected edges between protein nodes. By using the probabilistic condition shown in Equation (5) into Equation (6), we obtained the PMDS, formalized as the integer linear programming (ILP) described below.

### 4.6. ILP-Formalized PMDS Problem

Minimize $\sum_{v_{i}\in \;V}x_{i}$

 subject to:

(8) $$\begin{array}{c} x_{j}\geq 1,\;\forall \;v_{j}\in V\;\mathrm{such}\;\mathrm{that}\;\deg(v_{j})=0,\\ -\ln(1-\Theta )x_{j}+\sum_{(v_{i},v_{j})\in E}(-\ln(\rho_{ij})x_{i})\geq -\ln(1-\Theta ),\;\forall \;v_{j}\in V\;\mathrm{such}\;\mathrm{that}\;\deg(v_{j})>0 \end{array}$$

where $\deg(v_{j})$denotes the degree of the protein node $v_{j}$. In the above equation, the first term $-\ln(1-\Theta )x_{j}$ is added because if node $v_{j}$is included in the PMDS, the inequality needs to hold.

### 4.7. Critical Probabilistic Control Model (CPMDS) and Its Efficient Algorithm

The above model identifies the minimum dominating set of proteins that can control the entire network with at least Θ probability. However, because the MDS solution, in general, is not unique, the above PMDS also does not give a unique solution. There are many potential sets of proteins that can cover the remaining proteome. As in previous works, we needed to classify the nodes into several control categories, namely, critical, intermittent and redundant nodes. Efficient algorithms that identify these categories in directed and undirected networks in the context of the MDS controllability problem have already been proposed [20,21]. However, the PMDS problem is much more complex because the edges are weighted and probabilistic.

In this study, we proposed a new CPMDS algorithm that can efficiently identify the control categories in a PMDS problem, which was our main computational result. The algorithm used three mathematical propositions that were applied as preprocessing steps on the network structure and lead to pre-identification of the control category of a fraction of the nodes. As a result, the remaining reduced network could be solved faster. The algorithm not only reduced the computational time but also expanded the computable network’s size.

The three newly proposed propositions were as follows:

**Proposition** **1.** *If a node *$v_{i}$*has two or more neighbours *$v_{j}$*with degree k = 1 and *$1-\rho_{ij}>\Theta$*holds, *$v_{i}$*is a critical node*.

**Proposition** **2.** *If all neighbours* $v_{j}$*of a node* $v_{i}$*are critical and* $1-\prod_{v_{j}\in N(v_{i})}\rho_{ij}>\Theta$*holds,* $v_{i}$*is a redundant node*.

**Proposition** **3.** *If* $v_{i}$*satisfies* $1-\prod_{v_{j}\in N(v_{i})}\rho_{ij}<\Theta$*,*$v_{i}$*is a critical node*.

The proposed algorithm combined these new propositions with the existing PMDS algorithm to identify the control categories as follows:

1. We applied Proposition 1 for each node.
2. We applied Proposition 2 for each remaining node.
3. We applied Proposition 3 for each remaining node.

Note that by preprocessing steps 1, 2, and 3, a subset of critical and redundant nodes was determined together with a lower bound |PM*_L_*| of the PMDS without performing any ILP computation.

4. We identified the control category for the remaining unclassified nodes by applying the algorithmic procedure proposed in Ref. [19] (Nacher and Akutsu, 2014). However, instead of solving an MDS, we solved a PMDS problem each time, as defined above (see also Figure 2).

4.1. We solved the PMDS problem for the simplified network after including information from 1~3 steps into the ILP.

4.2. For each node that belonged to the PMDS and was not already classified as critical, we identified its critical role by applying the critical procedure [19].

4.3. For each node that did not belong to the PMDS, we determined its redundant role by applying the redundant procedure [19].

Note that step 4.2 involved adding a constraint for a node that we knew belonged to at least one PMDS, such that the node was assumed to not be in a PMDS. For example, node 1 in Figure 2 belonged to a PMDS, but we imposed the opposite constraint *x_1_ =* 0. Next, we again computed the PMDS, and if the size increased, it was because *x_1_* was a critical node. Figure 2 also shows an example of step 4.3 in the identification of the remaining redundant nodes.

Figure 3 illustrates that the algorithm identified control categories for a large fraction of nodes despite the CPMDS being an NP-hard problem and the edges being probabilistic, which is remarkable. The algorithm was then applied to each constructed dynamic weighted network at different ages (see Figure 3).

### 4.8. Enrichment Calculation for Control Categories

The enrichment level of proteins associated with well-known ageing genes *A* that also appear in a given PMDS controllability category set *S* (critical, redundant or intermitted) was calculated as follows. First, we calculated the fraction of the number of nodes (proteins) associated with ageing genes ($N_{A}$) in the entire network of size *N* as $f_{A}=N_{A}/N$. Next, the fraction of the number of proteins associated with ageing gene *A* that appeared in a critical, redundant or intermittent set ($N_{A}^{S}$) of size $N^{S}$was calculated as $f_{A}^{S}=N_{A}^{S}/N^{S}$. Finally, the enrichment of proteins in an ageing gene set *A* for a critical, redundant or intermittent set *S* was computed as $E_{A}^{S}=\ln(f_{A}^{S}/f_{A})$.

### 4.9. Statistical Significance Tests

The exact two-tailed *p-value* for the enrichment of the PMDS critical control proteins in ageing genes was calculated using Fisher’s exact test. The complete results for all the brain regions and across the lifespan, for all 20-year increments and each Θ probability, are shown Figure 5. The results are displayed separately for female and male samples.

## Data Availability

We did not perform biological experiments, therefore we did not develop new biological data. The analysed gene expression data is publicly available from the GEO database file series GSE11882. The developed code for the main analysis can be found in the Supplementary Materials folder.

## Supplementary Materials

The Supplementary Materials are available online at https://www.mdpi.com/article/10.3390/ijms22189891/s1.

## References

[1] Szilard L. (1959). On the Nature of the Aging Process. Proc. Natl. Acad. Sci. USA.

[2] Melzer D., Pilling L.C., Ferrucci L. (2020). The genetics of human ageing. Nat. Rev. Genet..

[3] Berchtold N.C., Cribbs D.H., Zielke H.R., Cotman C.W., Coleman P.D., Rogers J., Head E., Kim R., Beach T., Miller C. (2008). Gene expression changes in the course of normal brain aging are sexually dimorphic. Proc. Natl. Acad. Sci. USA.

[4] Pakkenberg B. (2003). Aging and the human neocortex. Exp. Gerontol..

[5] Berchtold N.C., Coleman P.D., Cribbs D.H., Rogers J., Gillen D.L., Cotman C.W. (2013). Synaptic genes are extensively downregulated across multiple brain regions in normal human aging and Alzheimer’s disease. Neurobiol. Aging.

[6] Braak H., Braak E. (1991). Neuropathological stageing of Alzheimer-related changes. Acta Neuropathol..

[7] Liu Y.-Y., Slotine J.-J., Barabási A.-L. (2011). Controllability of complex networks. Nature.

[8] Nacher J.C., Akutsu T. (2012). Dominating scale-free networks with variable scaling exponent: Heterogeneous networks are not difficult to control. New J. Phys..

[9] Vinayagam A., Gibson T.E., Barabasi A., Lee H.-J., Yilmazel B., Roesel C., Hu Y., Kwon Y., Sharma A., Liu Y.-Y. (2016). Controllability analysis of the directed human protein interaction network identifies disease genes and drug targets. Proc. Natl. Acad. Sci. USA.

[10] Yan G., Vértes P.E., Towlson E.K., Chew Y.L., Walker D.S., Schafer W., Barabasi A. (2017). Network control principles predict neuron function in the Caenorhabditis elegans connectome. Nat. Cell Biol..

[11] Nacher J.C., Akutsu T. (2016). Minimum dominating set-based methods for analyzing biological networks. Methods.

[12] Lee B., Kang U., Chang H., Cho K.-H. (2019). The Hidden Control Architecture of Complex Brain Networks. Science.

[13] Wuchty S. (2014). Controllability in protein interaction networks. Proc. Natl. Acad. Sci. USA.

[14] Kagami H., Akutsu T., Maegawa S., Hosokawa H., Nacher J.C. (2015). Determining Associations between Human Diseases and non-coding RNAs with Critical Roles in Network Control. Sci. Rep..

[15] Sun P.G. (2015). Co-controllability of drug-disease-gene network. New J. Phys..

[16] Basler G., Nikoloski Z., Larhlimi A., Barabási A.-L., Liu Y.-Y. (2016). Control of fluxes in metabolic networks. Genome Res..

[17] Schwartz J.-M., Otokuni H., Akutsu T., Nacher J.C. (2019). Probabilistic controllability approach to metabolic fluxes in normal and cancer tissues. Nat. Commun..

[18] Jia T., Liu Y.Y., Csóka E., Pósfai M., Slotine J.J., Barabási A.L. (2013). Emergence of bimodality in controlling complex networks. Nat. Commun..

[19] Nacher J.C., Akutsu T. (2014). Analysis of critical and redundant nodes in controlling directed and undirected complex networks using dominating sets. J. Complex Netw..

[20] Ishitsuka M., Akutsu T., Nacher J.C. (2016). Critical controllability in proteome-wide protein interaction network integrating transcriptome. Sci. Rep..

[21] Ishitsuka M., Akutsu T., Nacher J.C. (2017). Critical controllability analysis of directed biological networks using efficient graph reduction. Sci. Rep..

[22] Viger F., Latapy M. Efficient and Simple Generation of Random Simple Connected Graphs with Prescribed Degree Sequence. Proceedings of the 11th International Conference Computing and Combinatorics Conference.

[23] Van Deursen J.M. (2014). The role of senescent cells in ageing. Nature.

[24] Cristofalo V.J., Lorenzini A., Allen R., Torres C., Tresini M. (2004). Replicative senescence: A critical review. Mech. Ageing Dev..

[25] Bodnar A.G., Ouellette M., Frolkis M., Holt S.E., Chiu C.-P., Morin G., Harley C.B., Shay J.W., Lichtsteiner S., Wright W.E. (1998). Extension of Life-Span by Introduction of Telomerase into Normal Human Cells. Science.

[26] Bär C., Blasco M.A. (2016). Telomeres and telomerase as therapeutic targets to prevent and treat age-related diseases. F1000Research.

[27] Chatterjee S. (2017). Telomeres in health and disease. J. Oral Maxillofac. Pathol..

[28] Epel E.S., Blackburn E.H., Lin J., Dhabhar F.S., Adler N.E., Morrow J.D., Cawthon R.M. (2004). Accelerated telomere shortening in response to life stress. Proc. Natl. Acad. Sci. USA.

[29] Zglinicki T., Martin-Ruiz C. (2005). Telomeres as Biomarkers for Ageing and Age-Related Diseases. Curr. Mol. Med..

[30] Whittemore K., Vera E., Martínez-Nevado E., Sanpera C., Blasco M.A. (2019). Telomere shortening rate predicts species life span. Proc. Natl. Acad. Sci. USA.

[31] Stelzer G., Rosen N., Plaschkes I., Zimmerman S., Twik M., Fishilevich S., Stein T.I., Nudel R., Lieder I., Mazor Y. (2016). The GeneCards Suite: From Gene Data Mining to Disease Genome Sequence Analysis. Curr. Protoc. Bioinform..

[32] Shin J.W., Kwon S.H., Choi J.Y., Na J.I., Huh C.H., Choi H.R., Park K.C. (2019). Molecular Mechanisms of Dermal Aging and Antiaging Approaches. Int. J. Mol. Sci..

[33] Labat-Robert J. (2004). Cell–matrix interactions in aging: Role of receptors and matricryptins. Ageing Res. Rev..

[34] Gautier L., Møller M., Friis-Hansen L., Knudsen S. (2004). Alternative mapping of probes to genes for Affymetrix chips. BMC Bioinform..

[35] Lu D., Mausel P., Brondízio E., Moran E. (2004). Change detection techniques. Int. J. Remote Sens..

[36] Faisal F.E., Milenković T. (2014). Dynamic networks reveal key players in aging. Bioinformatics.

[37] Kawakami Y. (2017). Investigation into Aging Mechanism with Topology Analysis of Dynamic Networks. Master’s Thesis.

[38] Das J., Yu H. (2012). HINT: High-quality protein interactomes and their applications in understanding human disease. BMC Syst. Biol..

[39] Tacutu R., Thornton D., Johnson E., Budovsky A., Barardo D., Craig T., Diana E., Lehmann G., Toren D., Wang J. (2018). Human Ageing Genomic Resources: New and updated databases. Nucleic Acids Res..

[40] Nacher J.C., Akutsu T. (2015). Structurally robust control of complex networks. Phys. Rev. E.

