# Supplementary material for: Probabilistic Critical Controllability Analysis of Protein Interaction Networks Integrating Normal Brain Ageing Gene Expression Profiles
Source: Int J Mol Sci. 2021 Sep 13;22(18):9891. doi: 10.3390/ijms22189891 (PMC8465977; doi:10.3390/ijms22189891)
Supplement: Supplementary file 1 [file ijms-22-09891-s001.zip › Supplementary_Materials.pdf]

Supplementary Materials

Probabilistic critical controllability analysis of protein interaction networks integrating normal brain ageing gene expression profiles

Eimi Yamaguchi, Tatsuya Akutsu & Jose C. Nacher

I. **Supplementary Information Figures S1-S33**
II. **Supplementary Information Table S1 and Tables S3-S6**
III. **Supplementary Information Excel Files:**
Excel Tables S2 and S7-S18 for the GO analysis can be downloaded electronically.

Enrichment calculation for each Gene Ontology annotation

The enrichment level of proteins in a given Gene Ontology (GO) annotation that are also classified into a given category set *S* was computed as follows. First, we compute the fraction of the number of proteins associated with a GO term *i* ($N_{GO(i)}$) in the whole network of size *N* as $f_{GO(i)} = N_{GO(i)} / N$. Then, the fraction of the number of proteins associated with a term $GO_{(i)}$ that occurred in a given set S ($N^S_{GO(i)}$) of size N^S was computed as $f^S_{GO(i)} = N^S_{GO(i)} / N^S$. Then, the enrichment of proteins in the term $GO_{(i)}$ for the given set *S* reads as: $E^S_{GO(i)} = \ln(f^S_{GO(i)} / f_{GO(i)})$. Each enrichment value was classified into one of three groups based on the GO term (biological process, molecular function or cellular component). Then, for all the enrichment values $E^C_{GO(i)}$ corresponding to the GO terms in each annotation class, a violin plot was constructed. Each split violin plot shows the probability density of data in its left half (male) and right half (female) for comparison. The results are shown in Fig. 9. Each protein was classified into five categories *S* based on whether it was engaged in critical (red), intermittent (yellow), redundant (blue) control or if it belonged to the well-known ageing set (green) or the overlapping of ageing genes and identified critical set (violet), the latter refer hereafter as the *critical ageing* category. P values for the associations between the critical ageing category and each specific gene ontology term are shown in SI Tables S2-S13 (Excel files). The results are displayed separately for each different age range, brain region and sex.

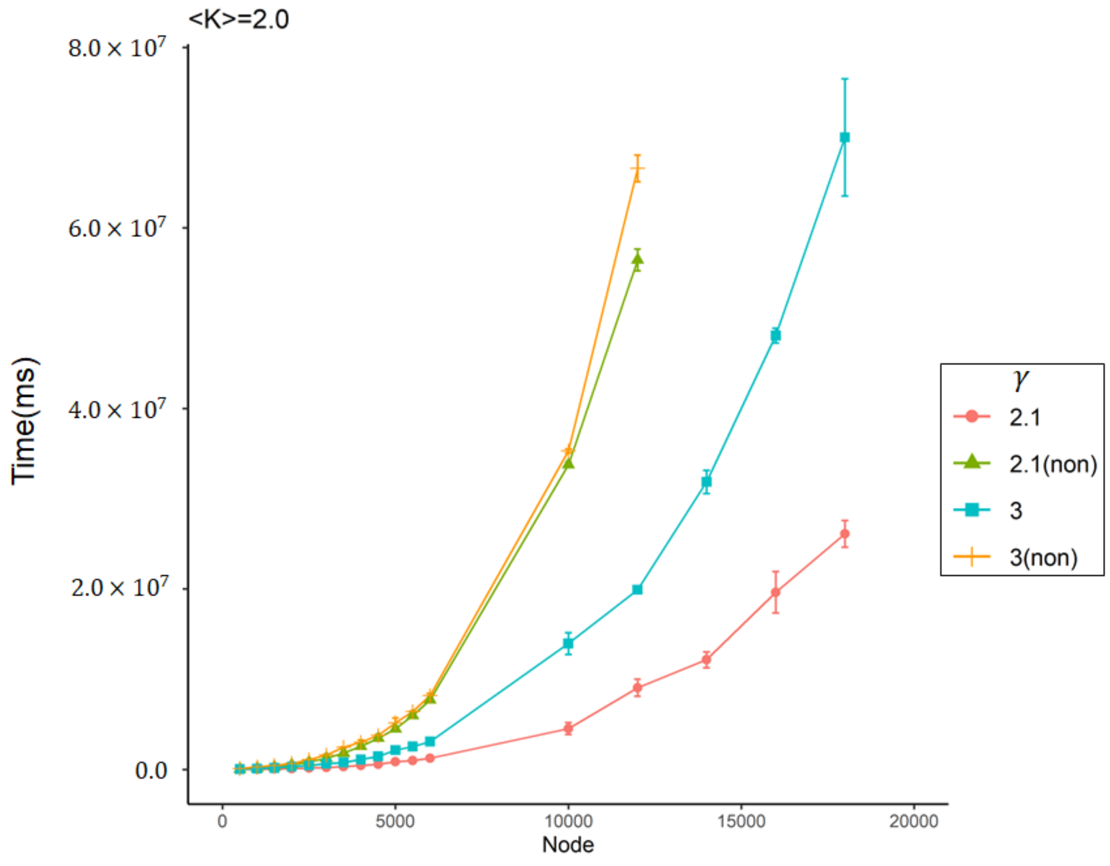

Fig. S1. Same as Fig. 4B but using linear scale.

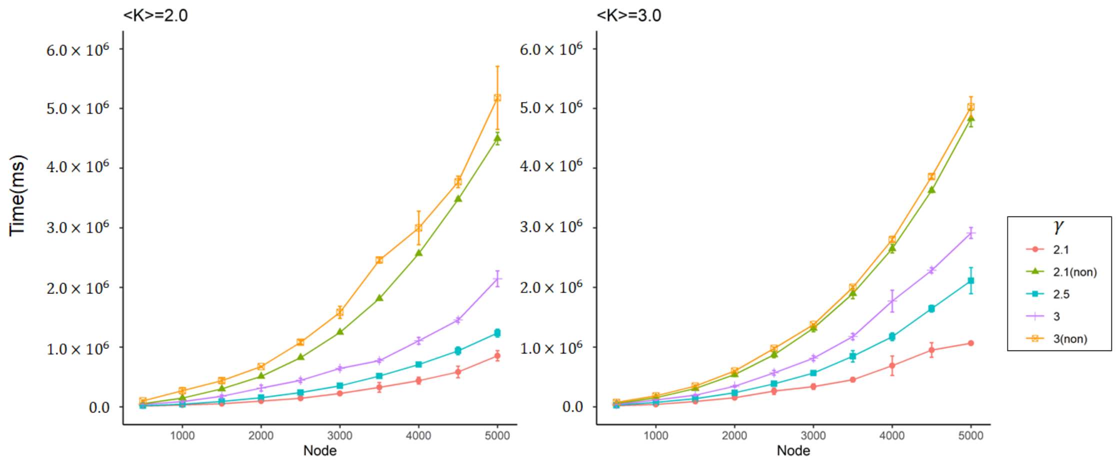

Fig. S2. Same as Fig. 4C but using linear scale.

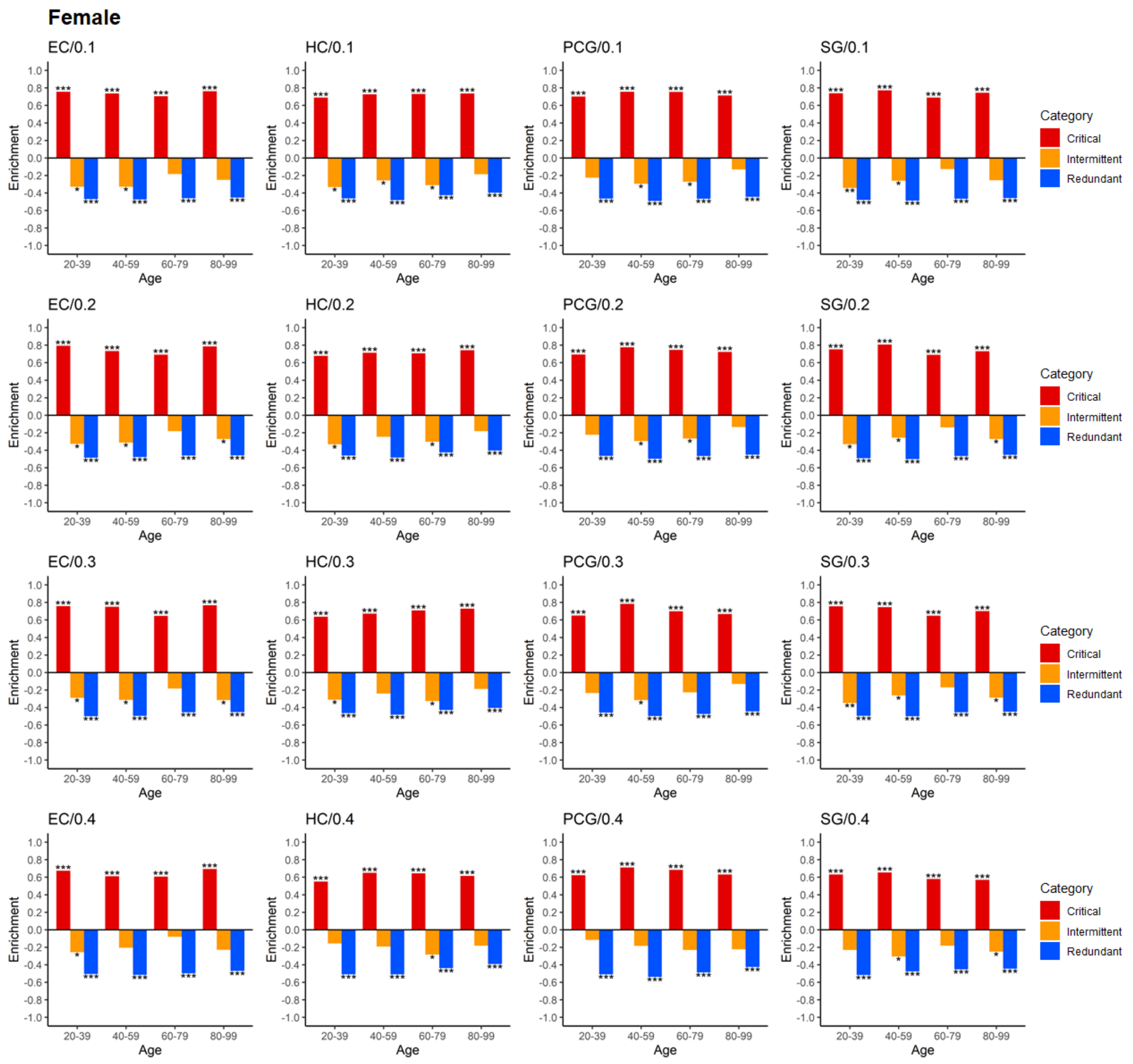

Fig. S3. Same as Fig. 6 but for different probabilistic threshold values Θ shown in figure

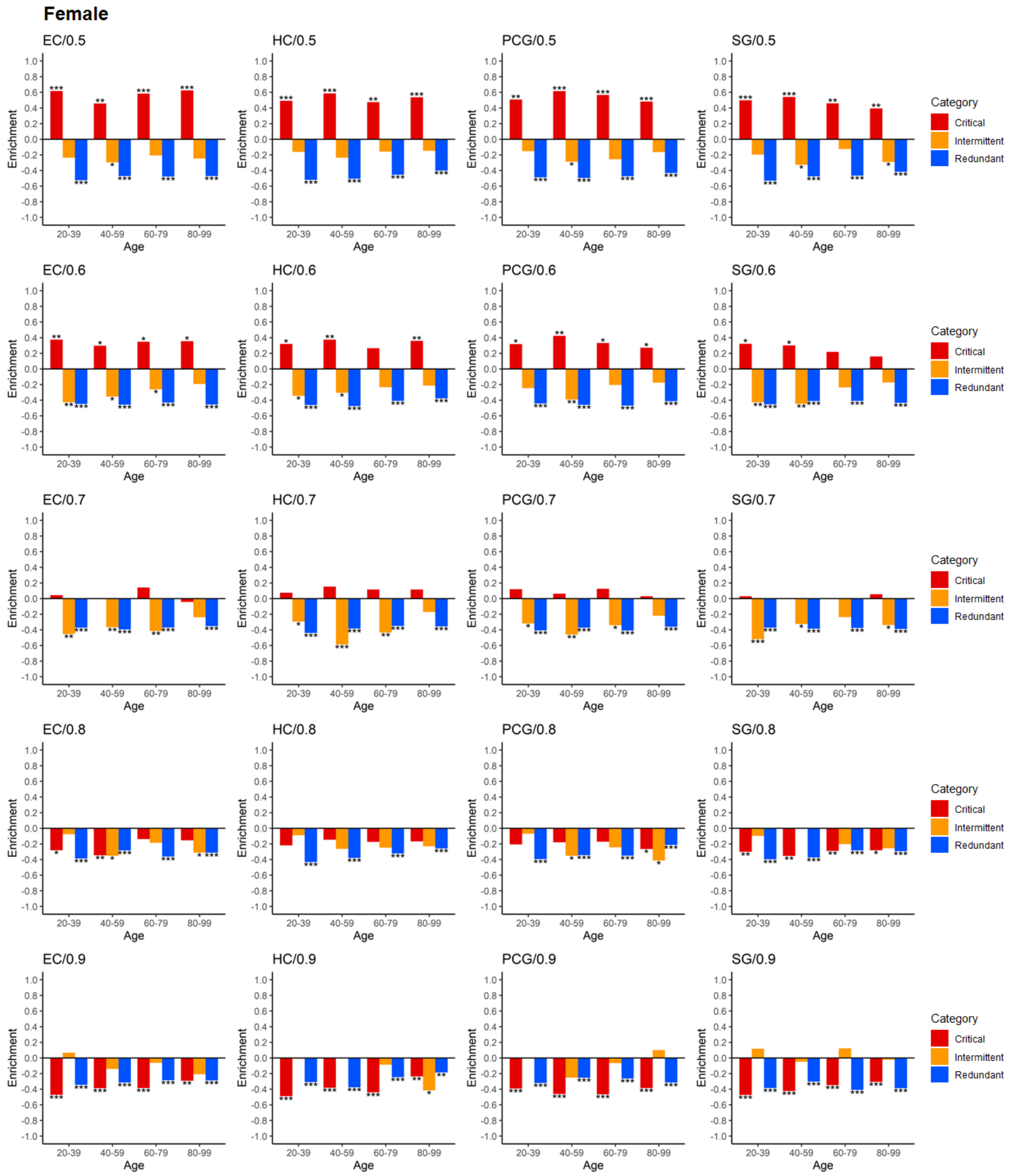

Fig. S4. Same as Fig. 6 but for different probabilistic threshold values Θ shown in figure.

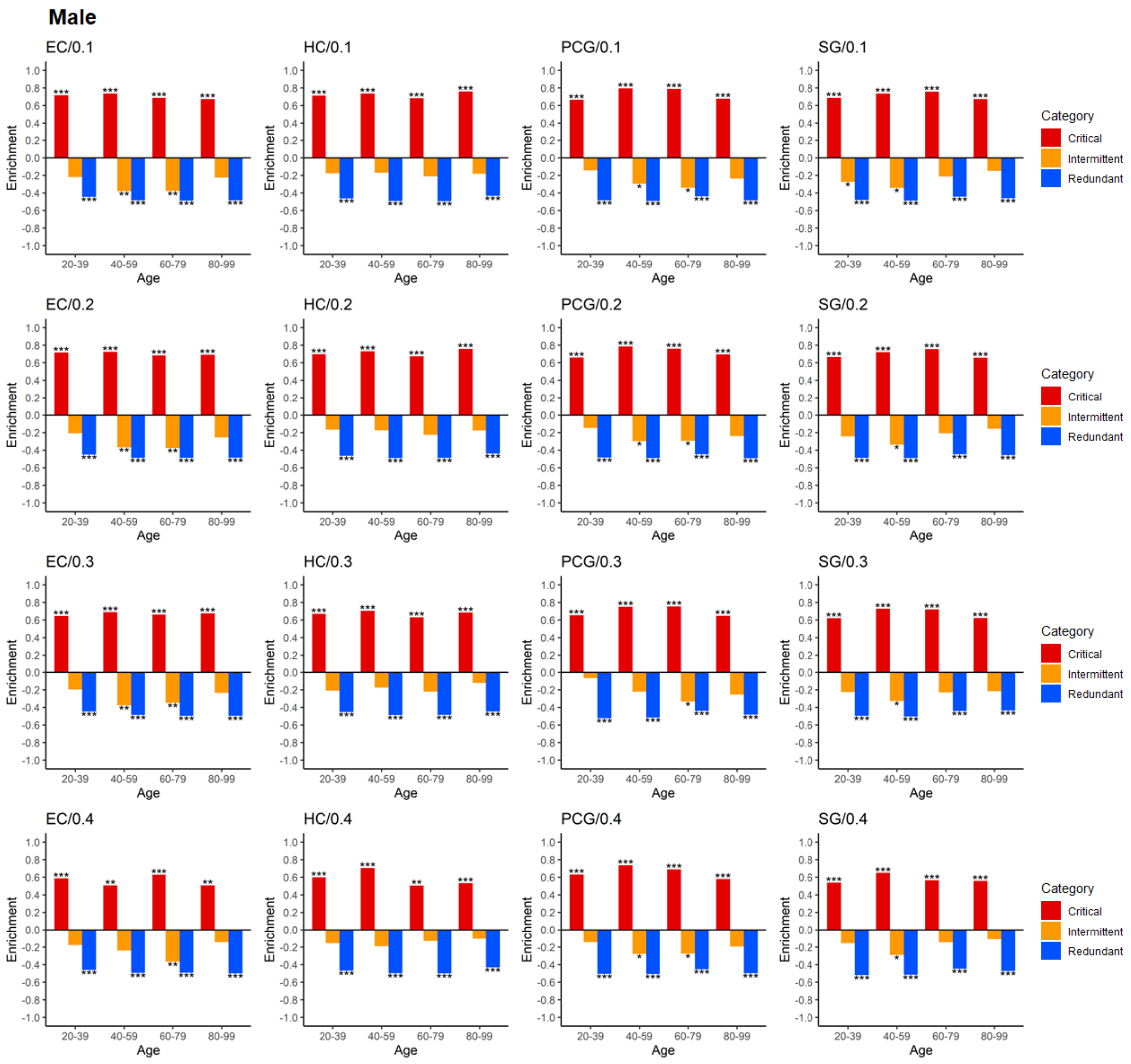

Fig. S5. Same as Fig. 6 but for male samples and different probabilistic threshold values Θ shown in figure

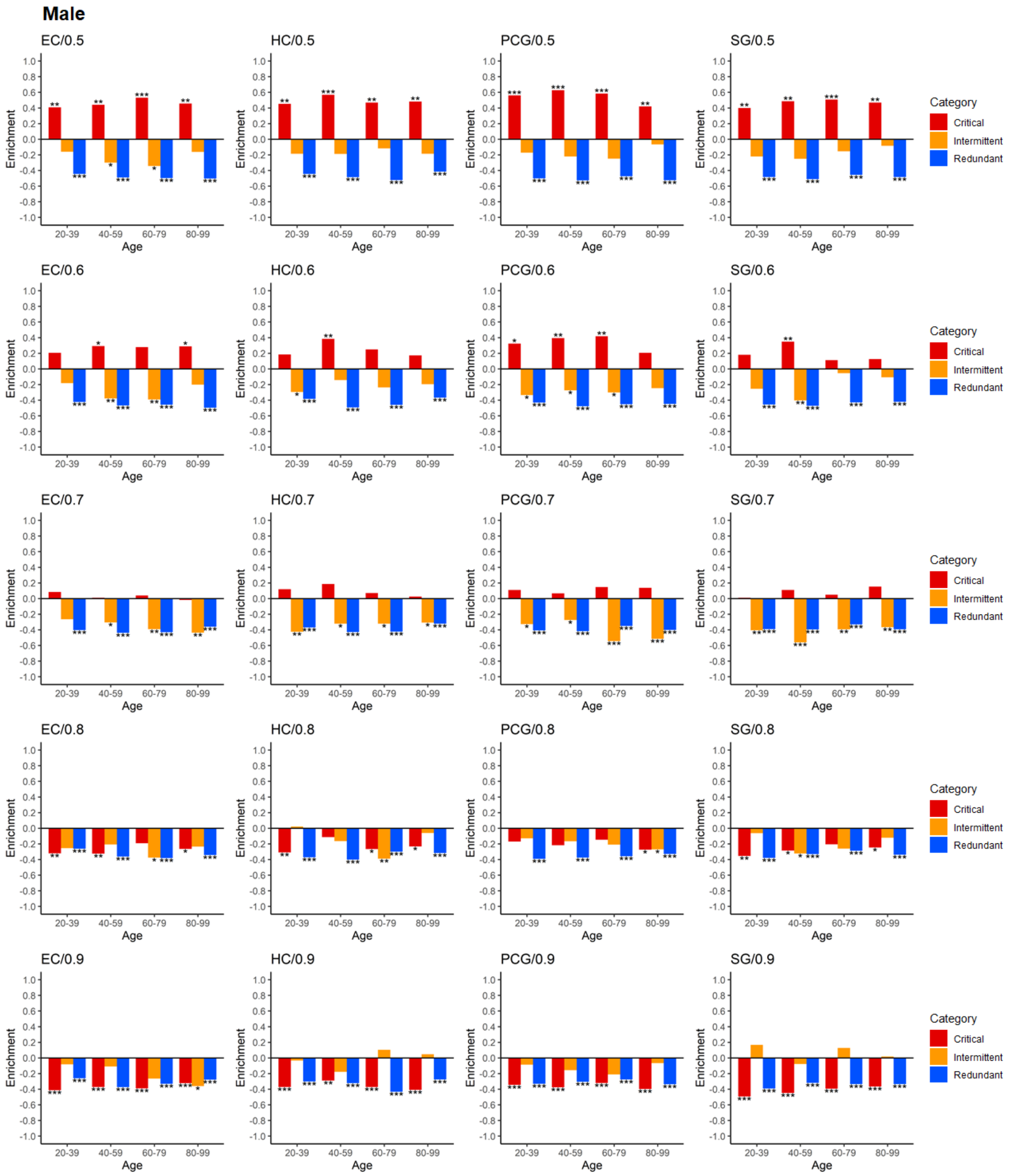

Fig. S6. Same as Fig. 6 but for male samples and different probabilistic threshold values Θ shown in figure

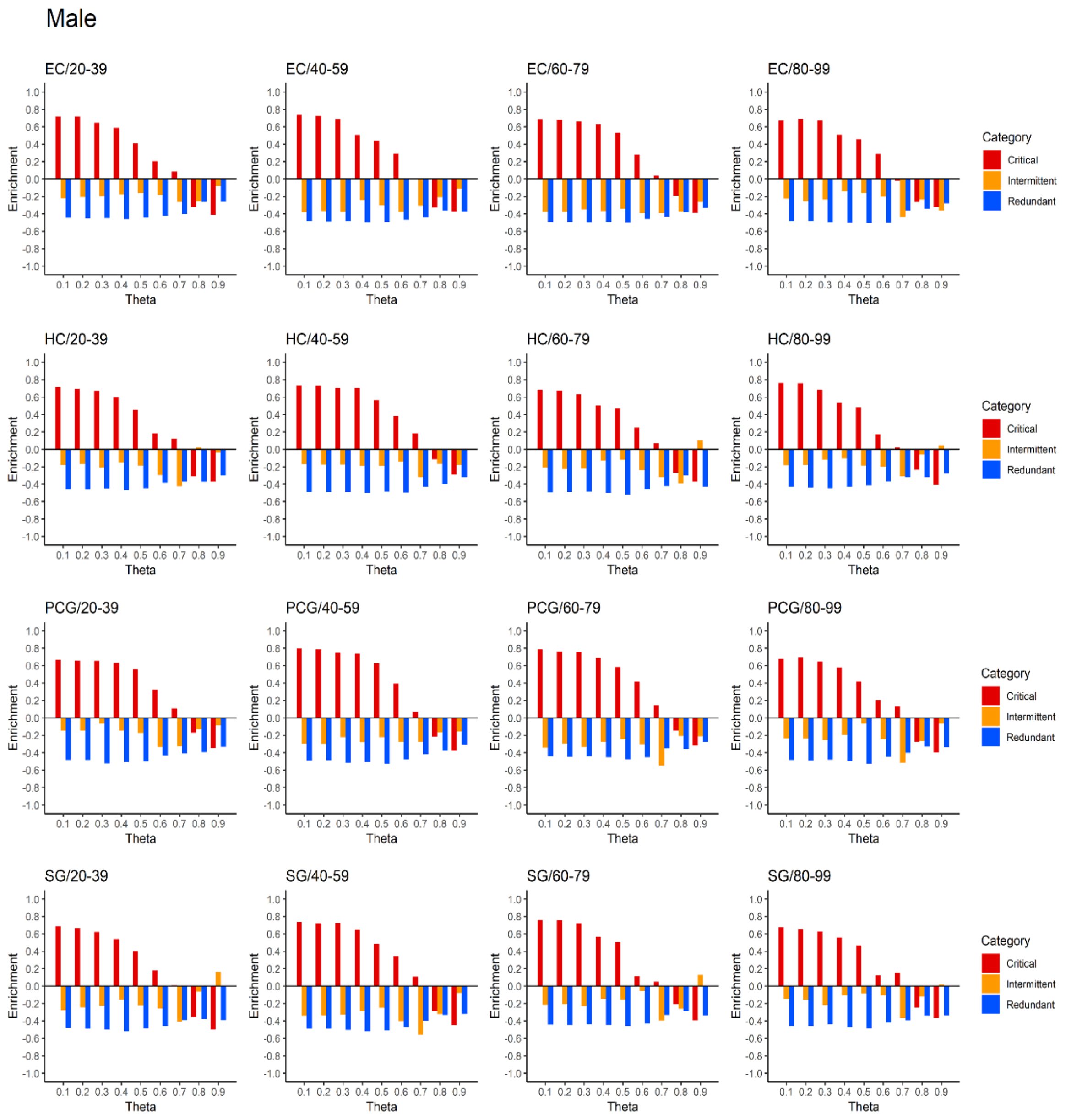

Fig. S7. Same as Fig. 7 but for male samples and different probabilistic threshold values Θ shown in figure. The associated P-values calculated using a two-tailed Fisher's exact test are shown in Table S2. (Excel file).

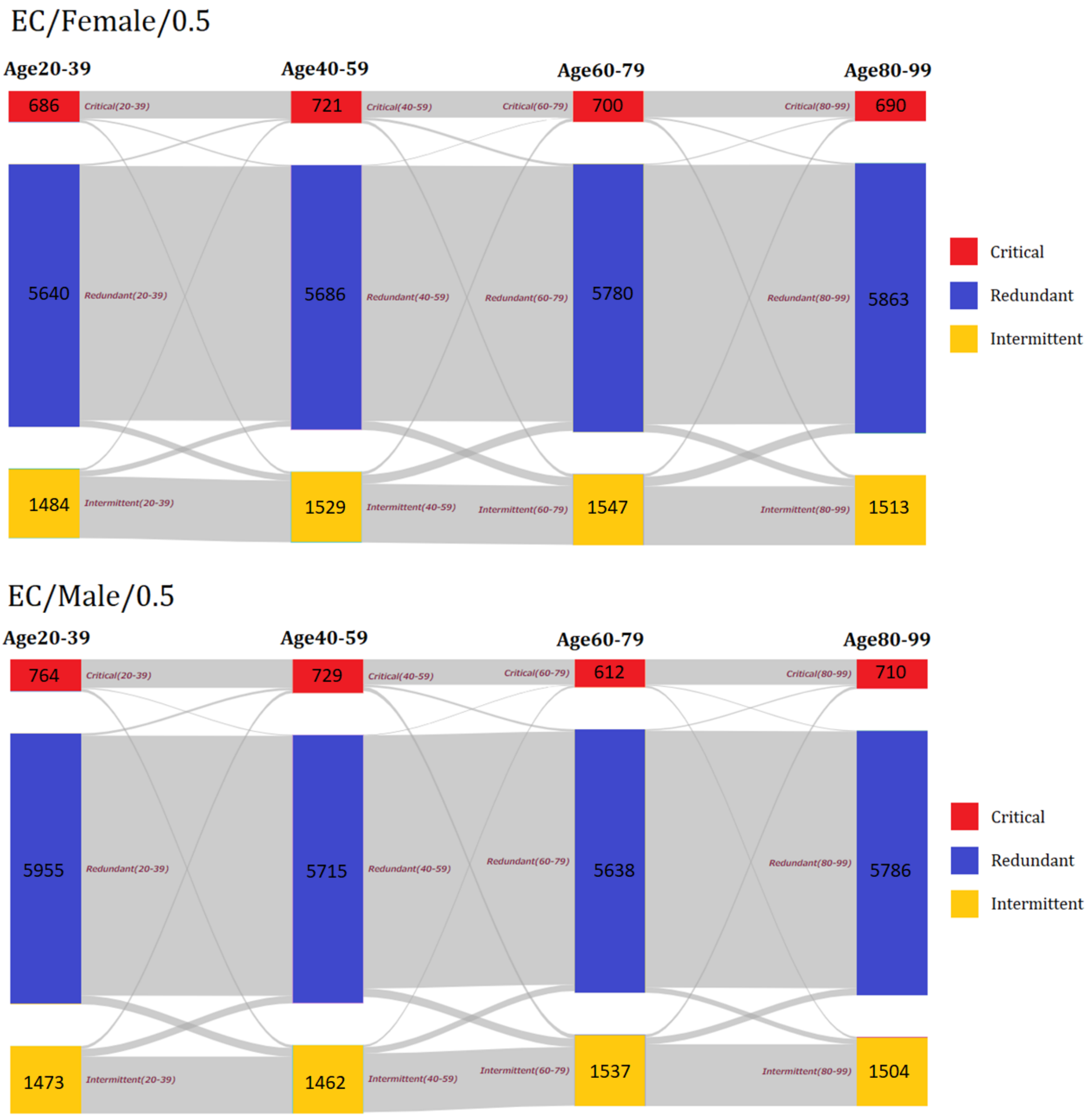

Fig. S8. The numbers of critical, intermittent and redundant genes that switch control category across lifespan for the EC brain region. The thickness of the grey lines is proportional to the number of proteins that change the control category.

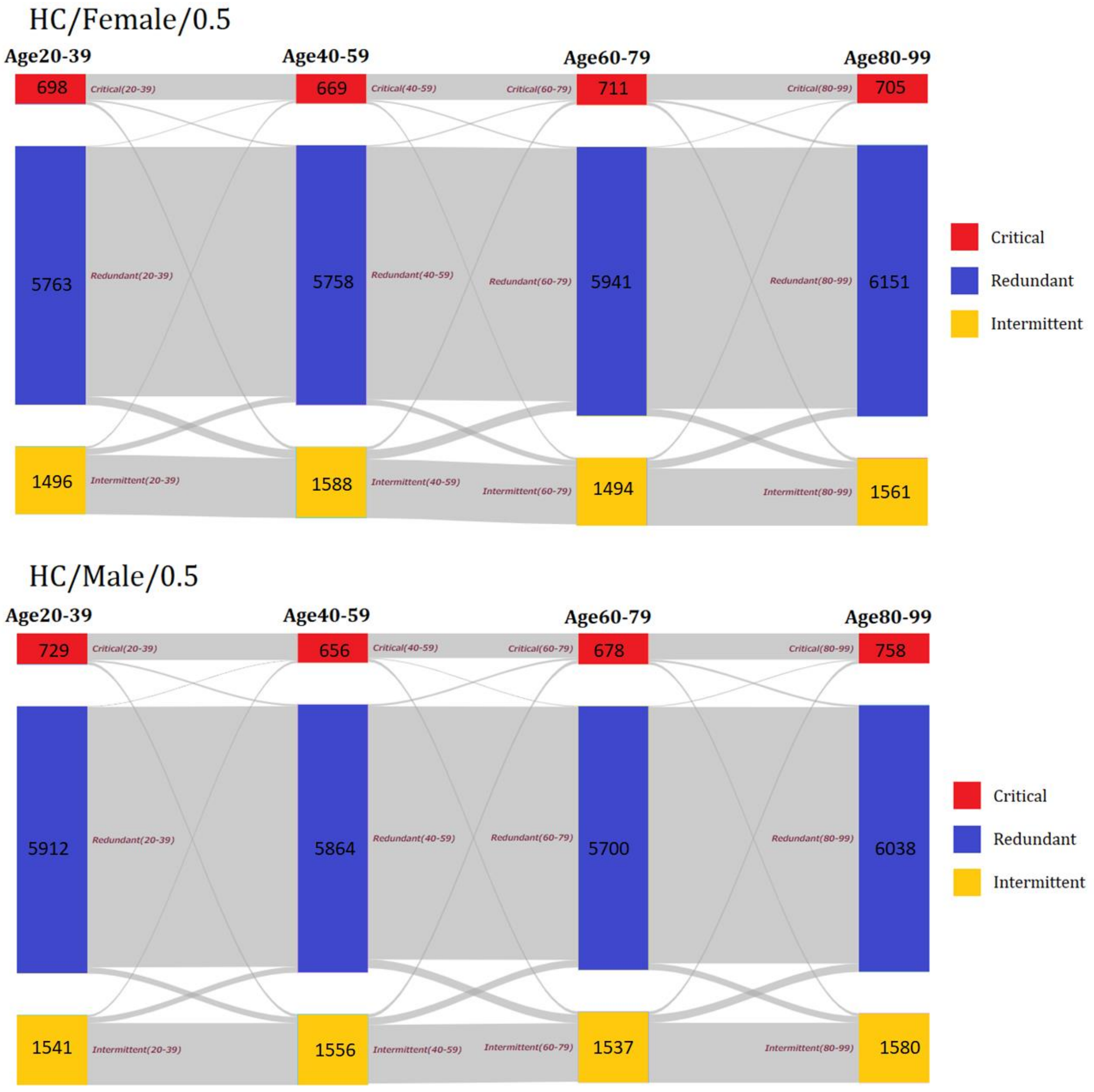

Fig. **S9**. The numbers of critical, intermittent and redundant genes that switch control category across lifespan for the HC brain region. The thickness of the grey lines is proportional to the number of proteins that change the control category.

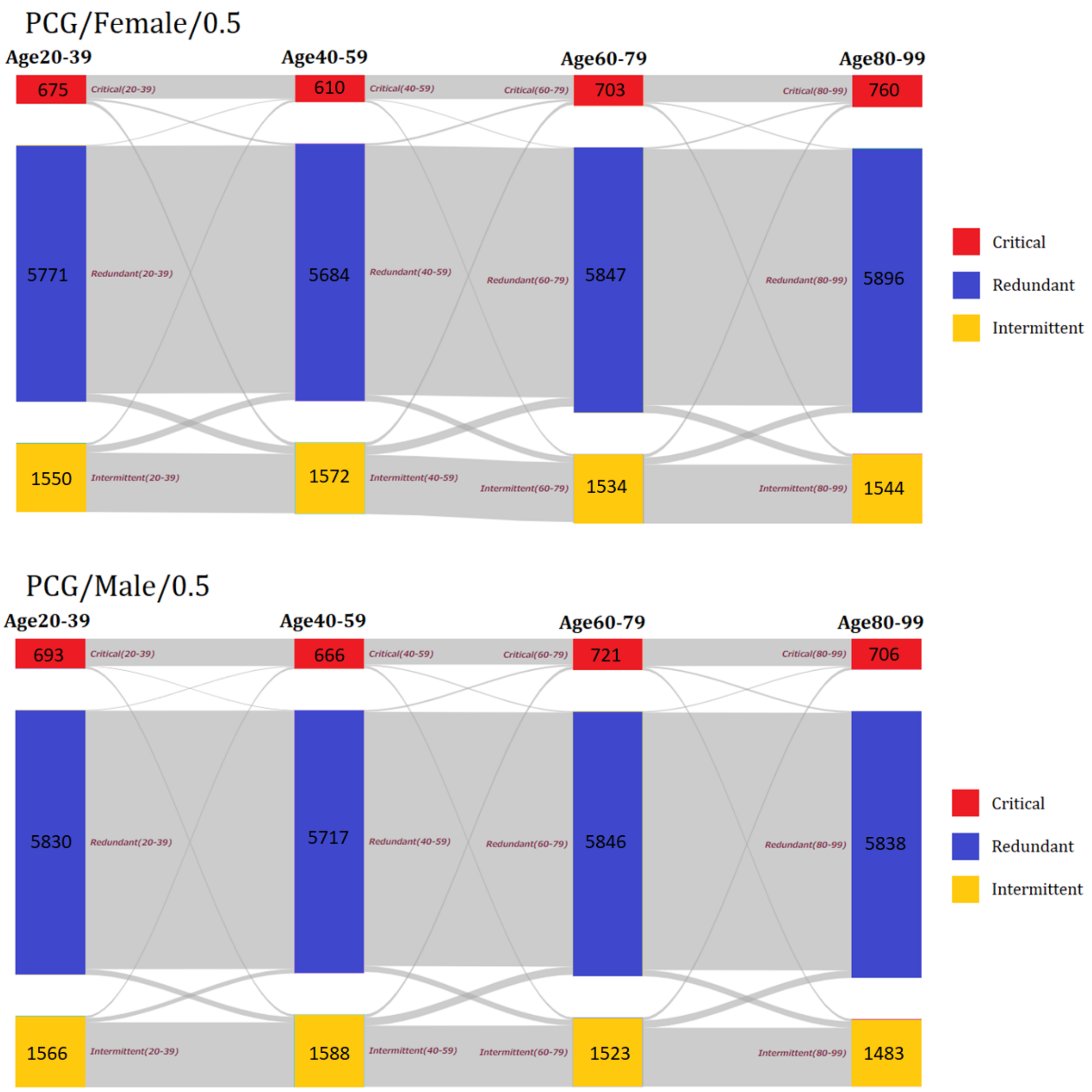

Fig. **S10**. The numbers of critical, intermittent and redundant genes that switch control category across lifespan for the PCG brain region. The thickness of the grey lines is proportional to the number of proteins that change the control category.

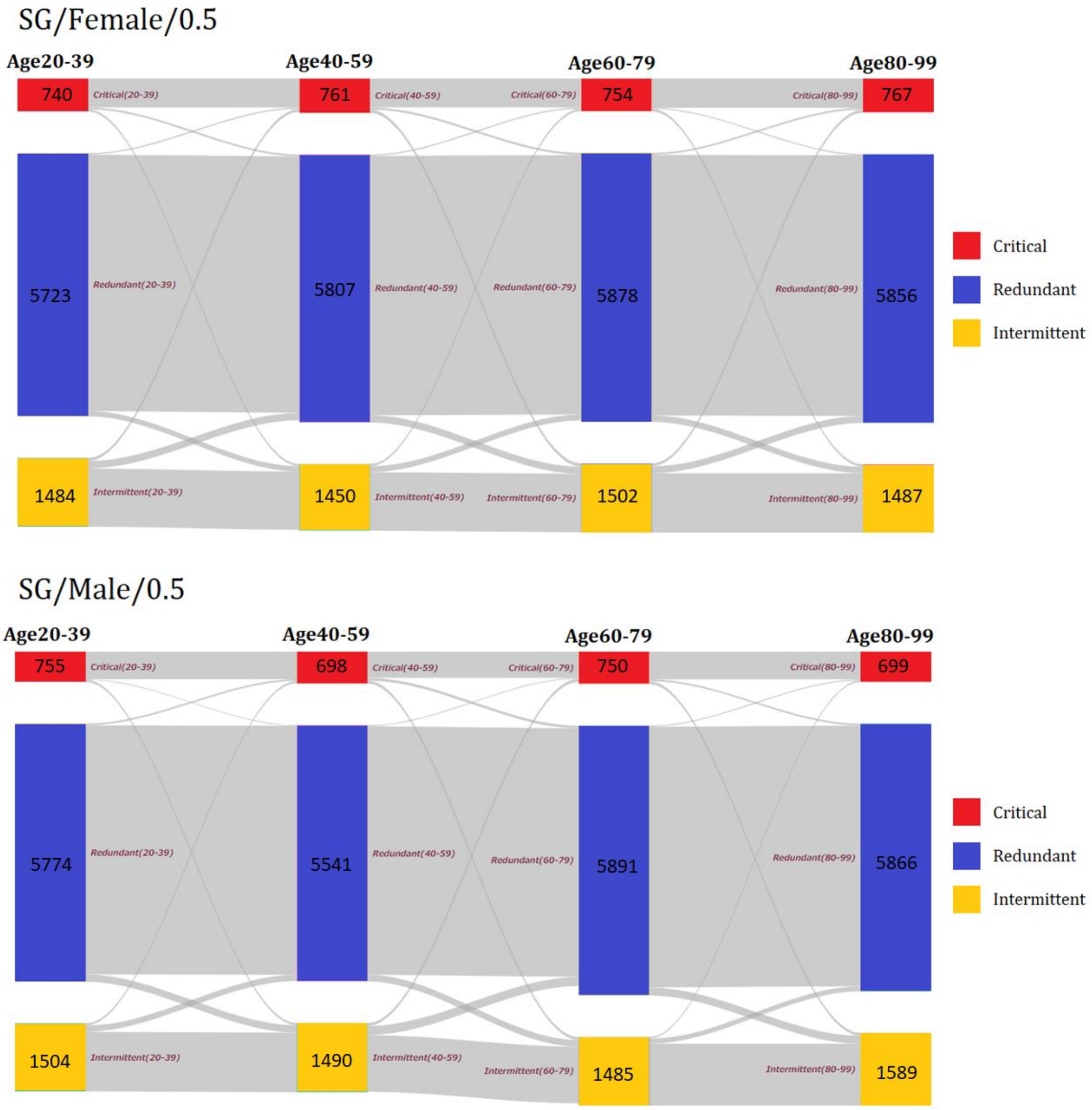

Fig. S11. The numbers of critical, intermittent and redundant genes that switch control category across lifespan for the SG brain region. The thickness of the grey lines is proportional to the number of proteins that change the control category.

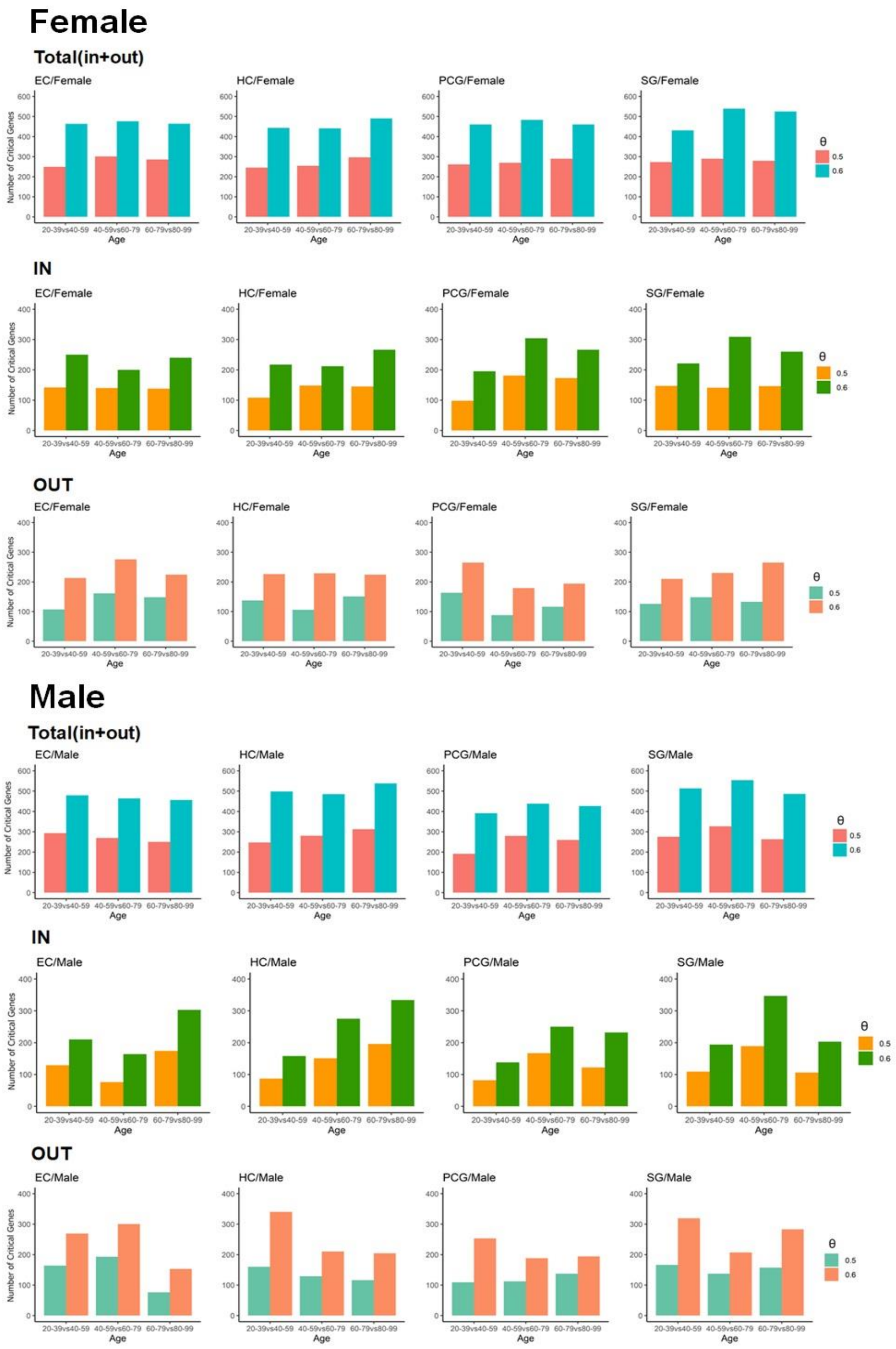

Fig. S12. Same as fig. 8 but showing results for both Θ=0.5 and 0.6.

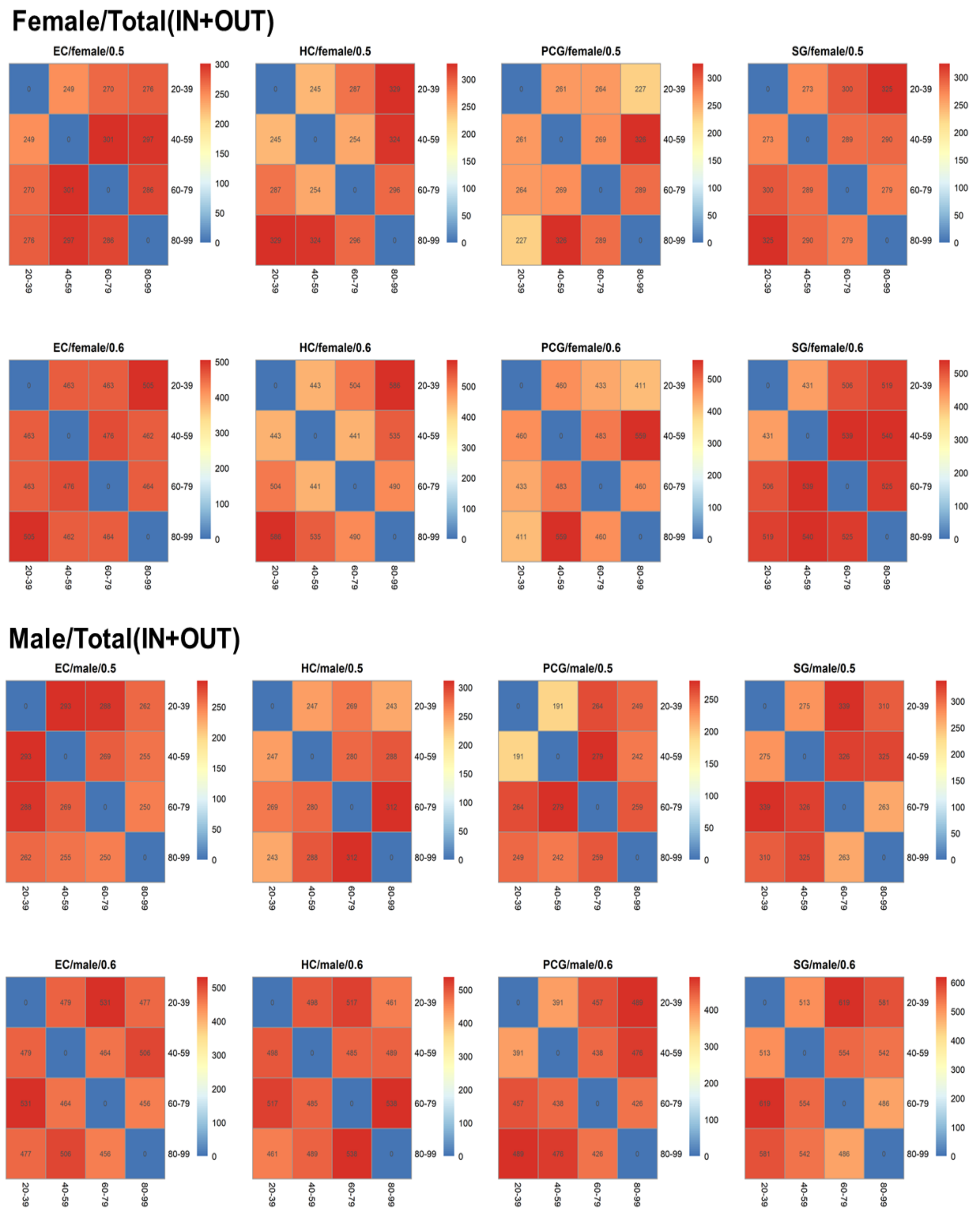

Fig. S13. A heat map plot that shows the change of all the critical proteins across different age range and different brain regions for Θ=0.5 and 0.6.

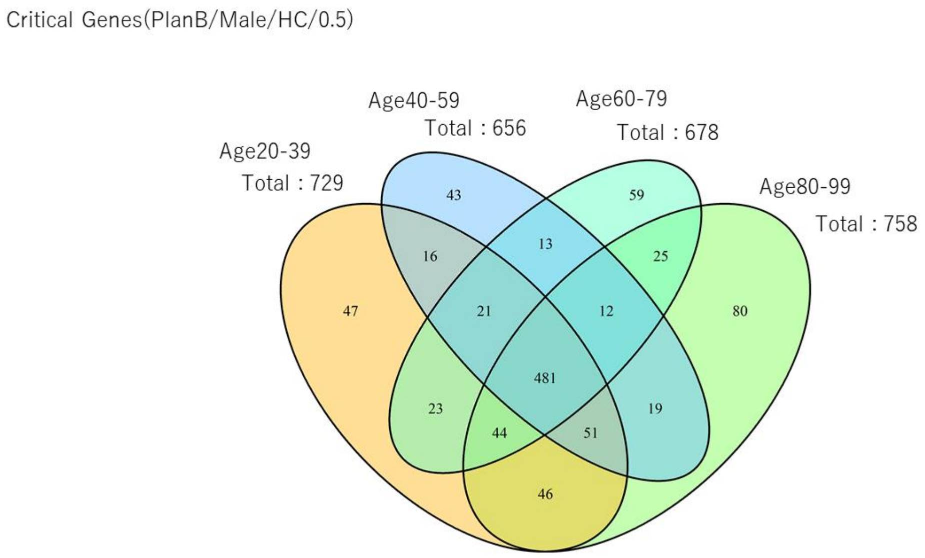

Fig. S14. The fraction of unique critical proteins at specific ages for male HC brain region samples and Θ=0.5.

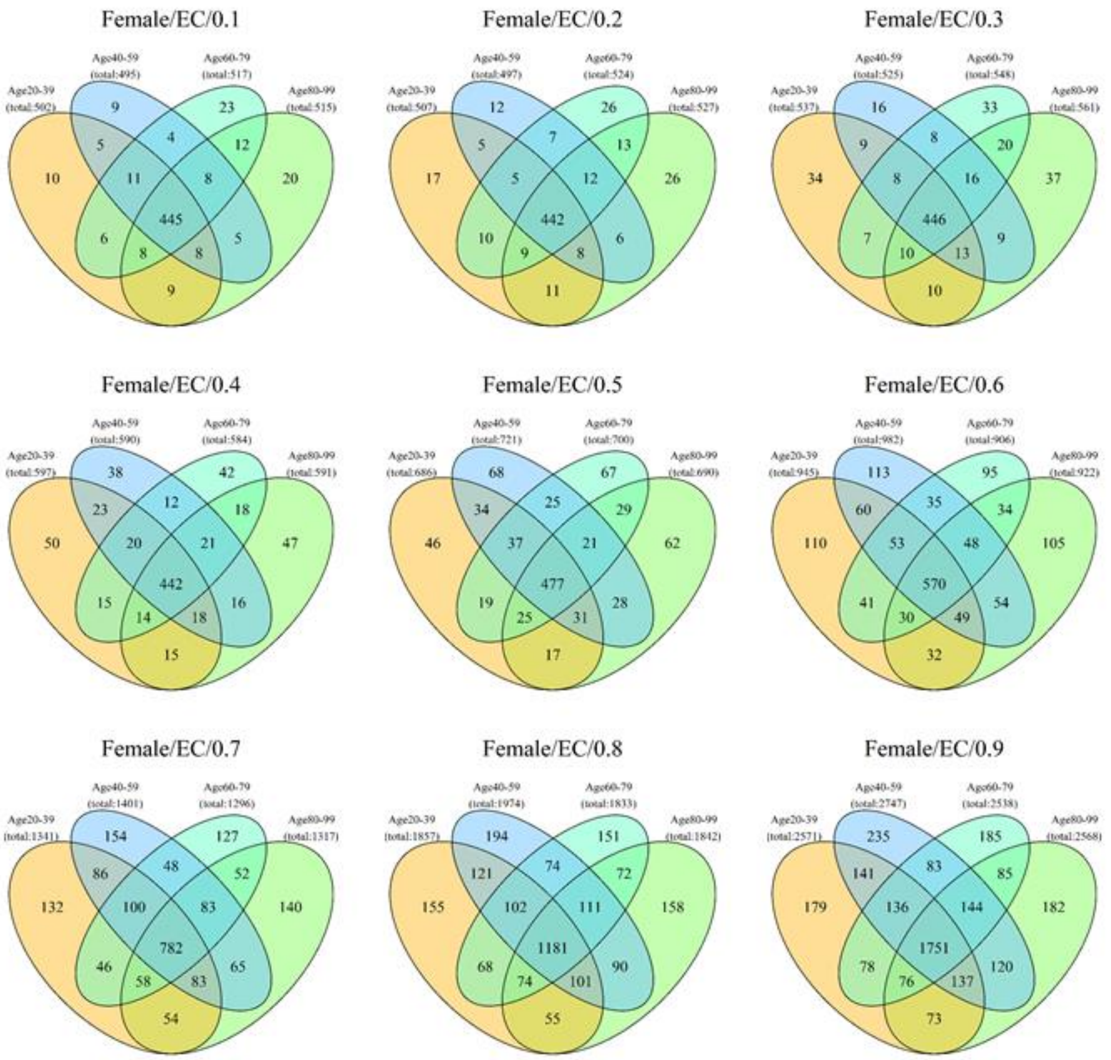

Fig. S15. The fraction of unique critical proteins at specific ages and for female EC brain regions samples and for each probabilistic threshold value Θ=0.5.

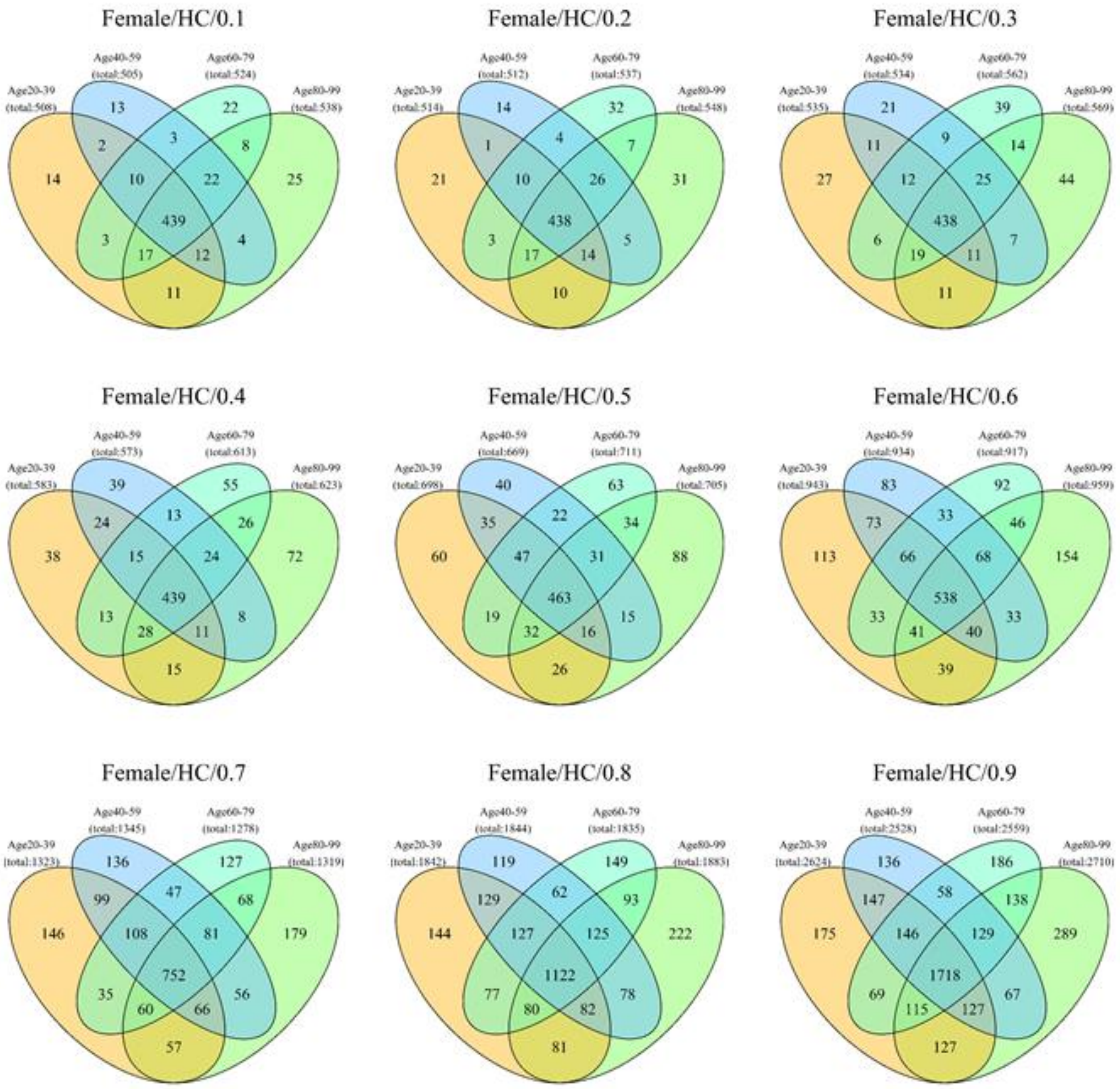

Fig. S16. The fraction of unique critical proteins at specific ages and for female HC brain regions samples and for each probabilistic threshold value Θ.

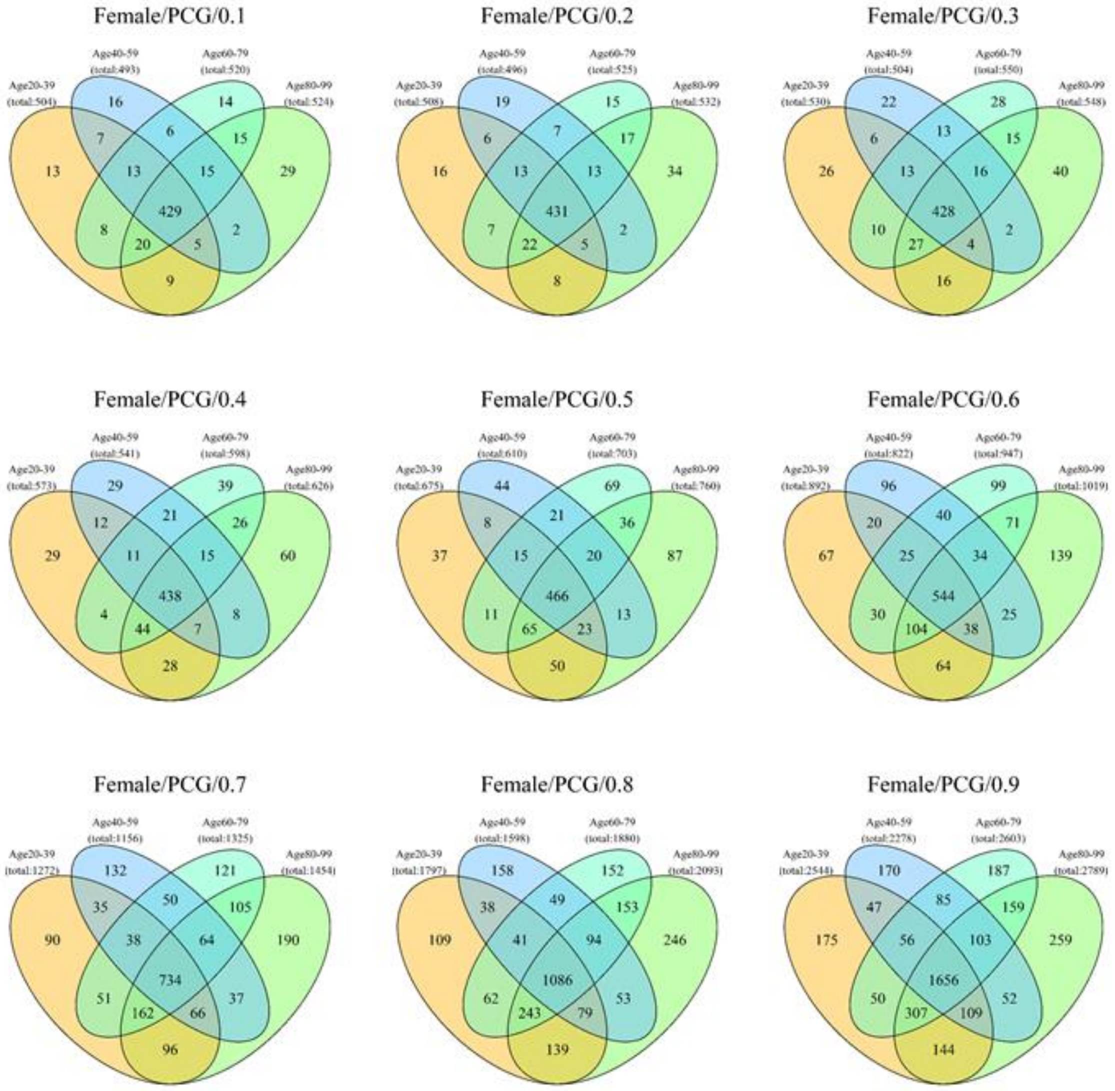

Fig. S17. The fraction of unique critical proteins at specific ages and for female PCG brain regions samples and for each probabilistic threshold value Θ.

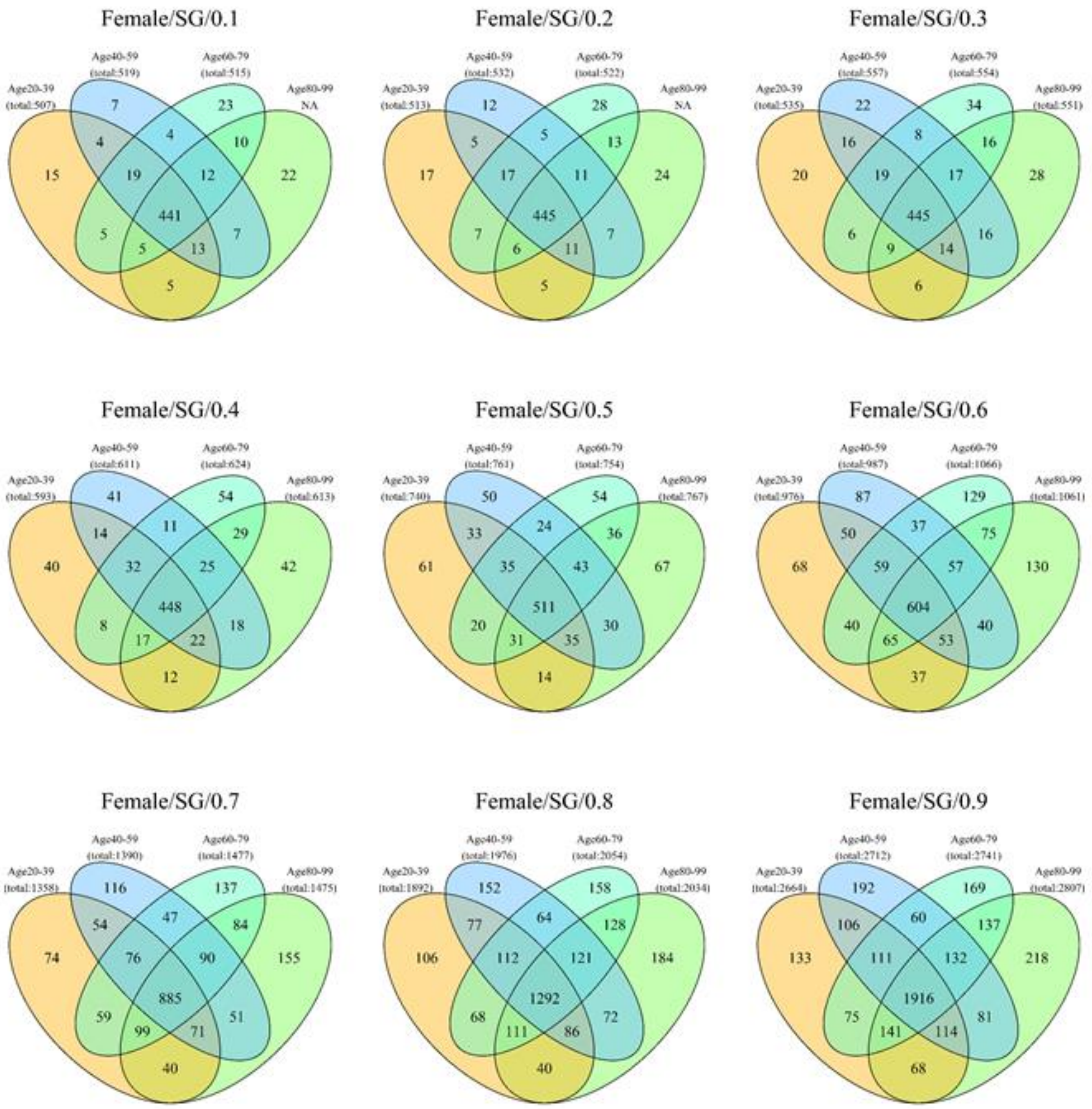

Fig. S18. The fraction of unique critical proteins at specific ages and for female SG brain regions samples and for each probabilistic threshold value Θ.

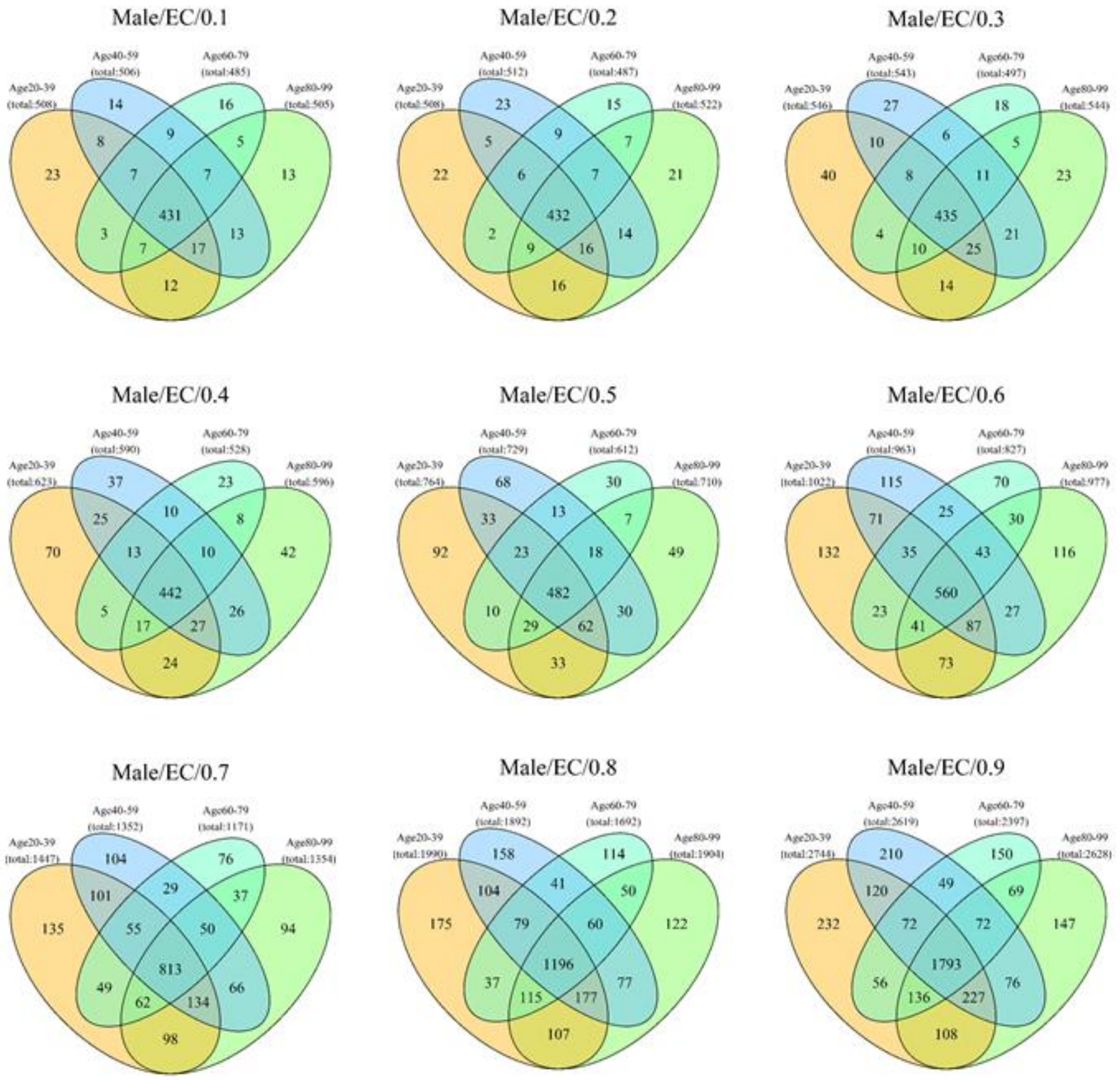

Fig. S19. The fraction of unique critical proteins at specific ages and for male EC brain regions samples and for each probabilistic threshold value Θ.

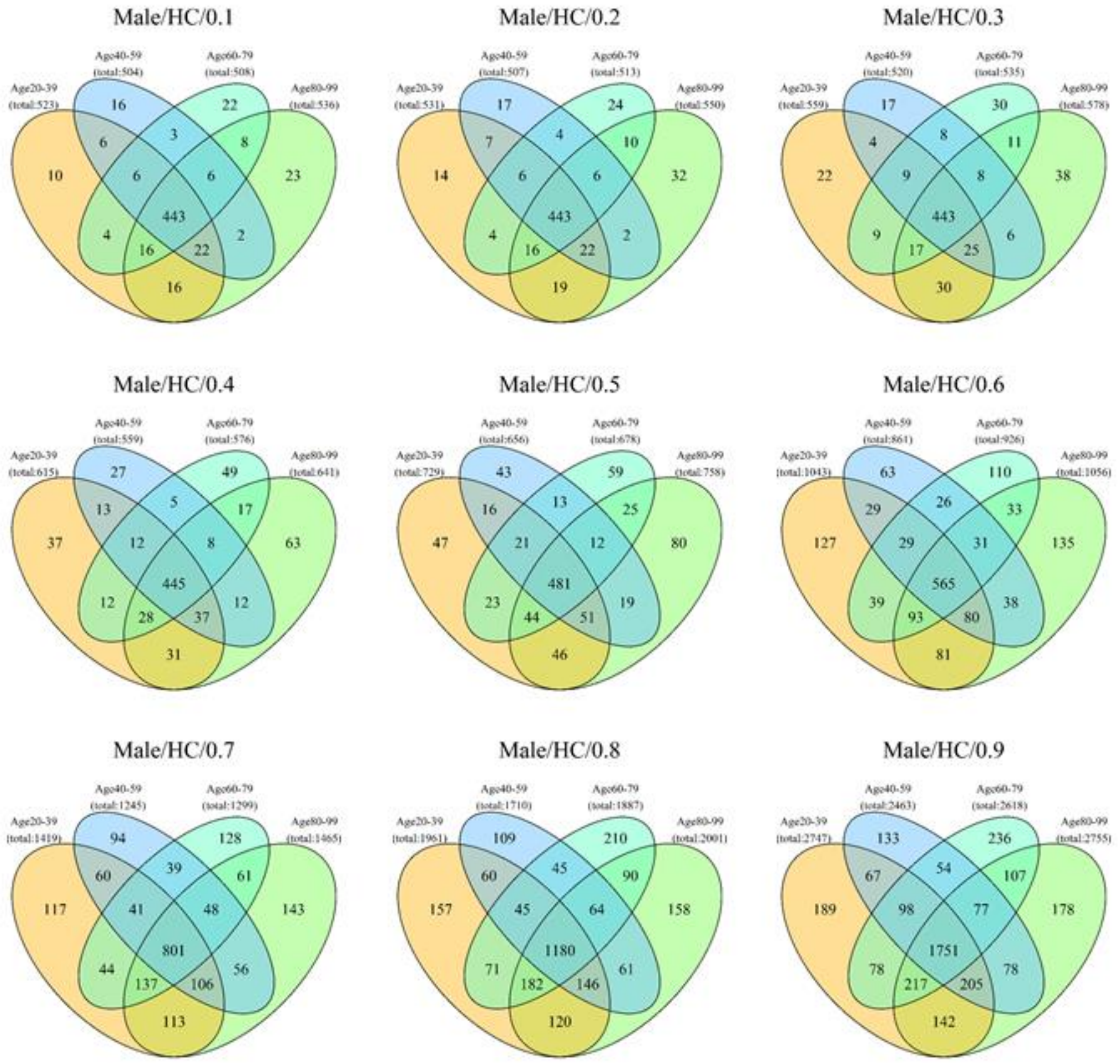

Fig. S20. The fraction of unique critical proteins at specific ages and for male HC brain regions samples and for each probabilistic threshold value Θ.

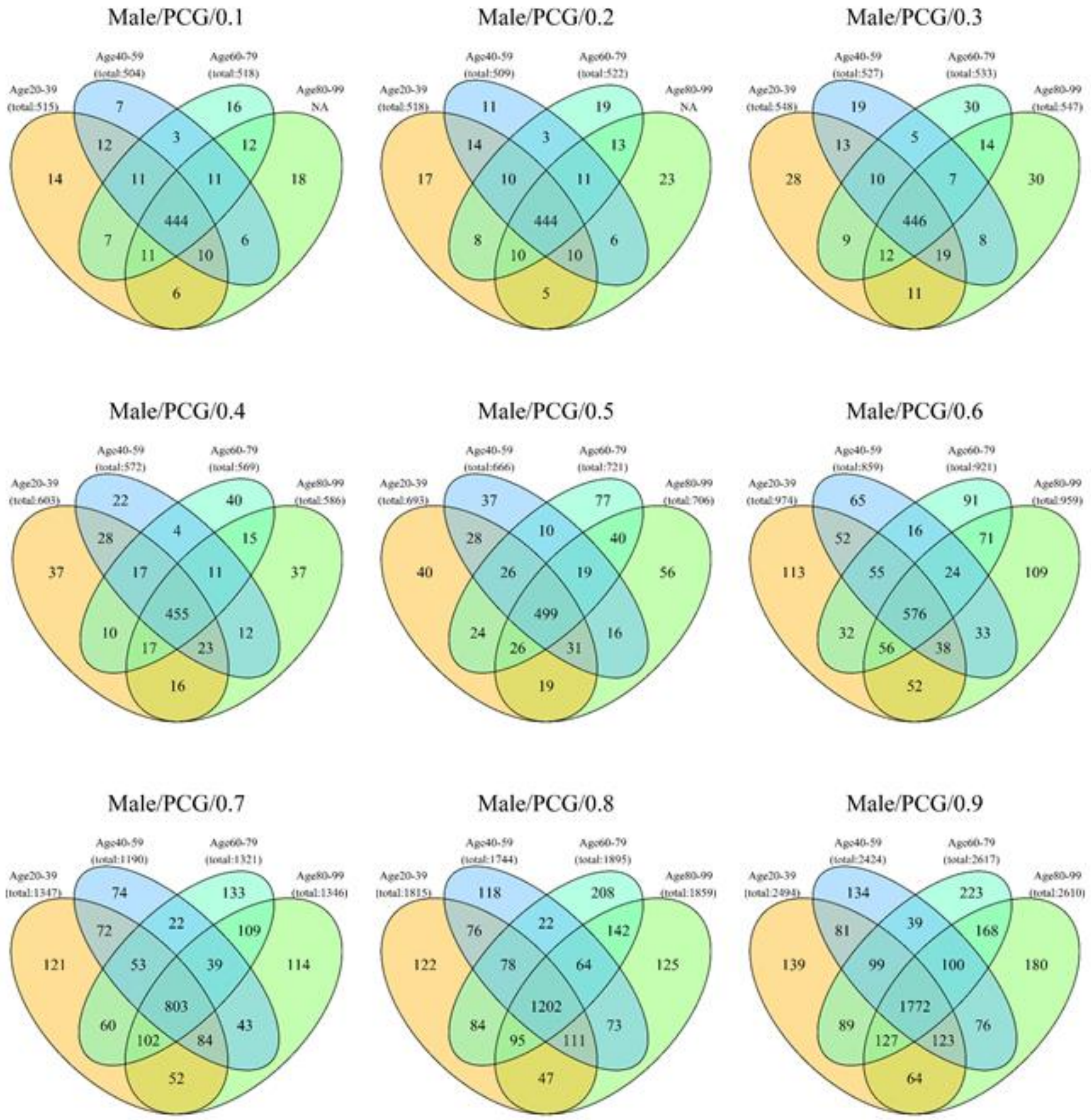

Fig. S21. The fraction of unique critical proteins at specific ages and for female PCG brain regions samples and for each probabilistic threshold value Θ.

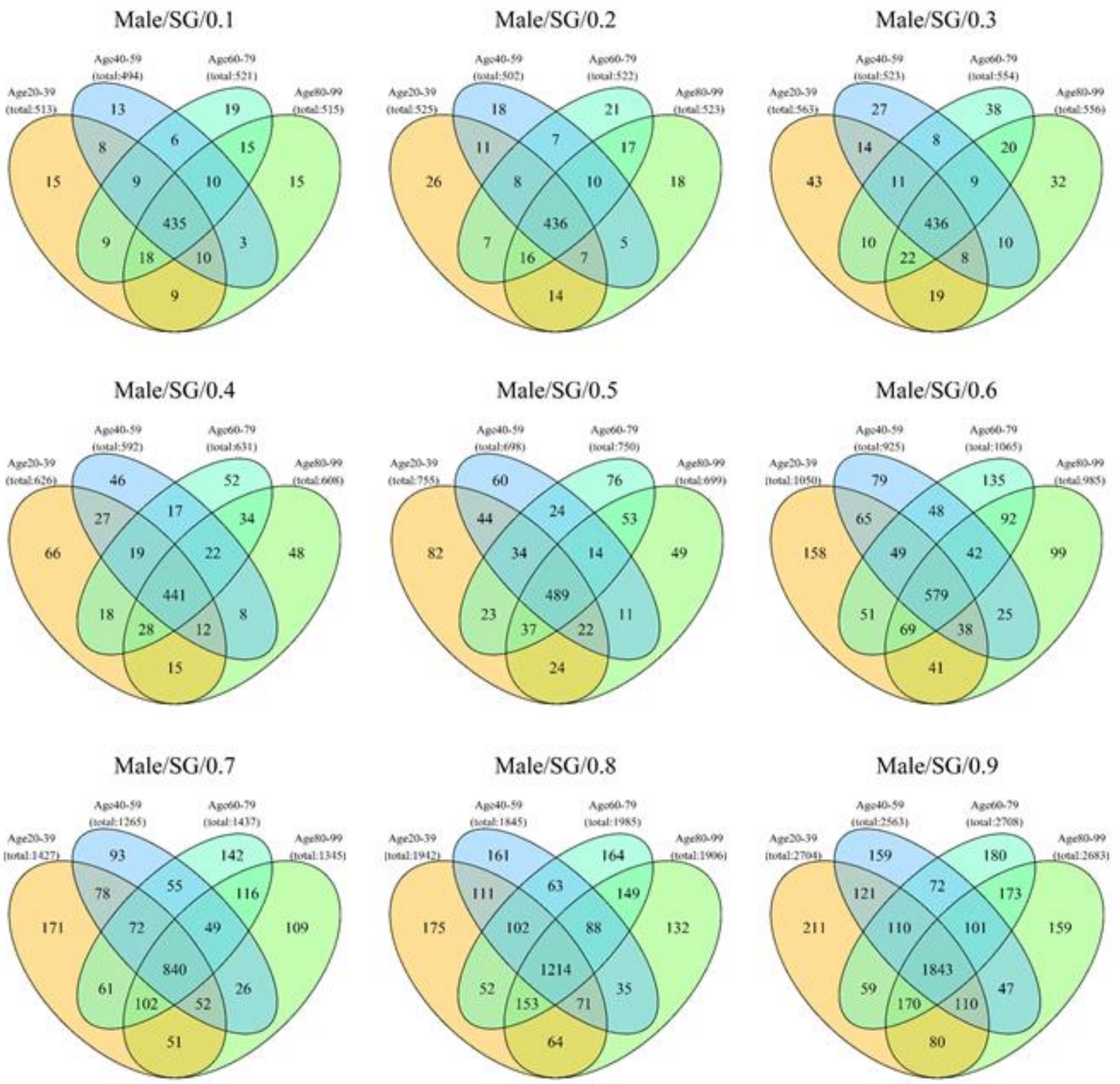

Fig. S22. The fraction of unique critical proteins at specific ages and for male SG brain regions samples and for each probabilistic threshold value Θ.

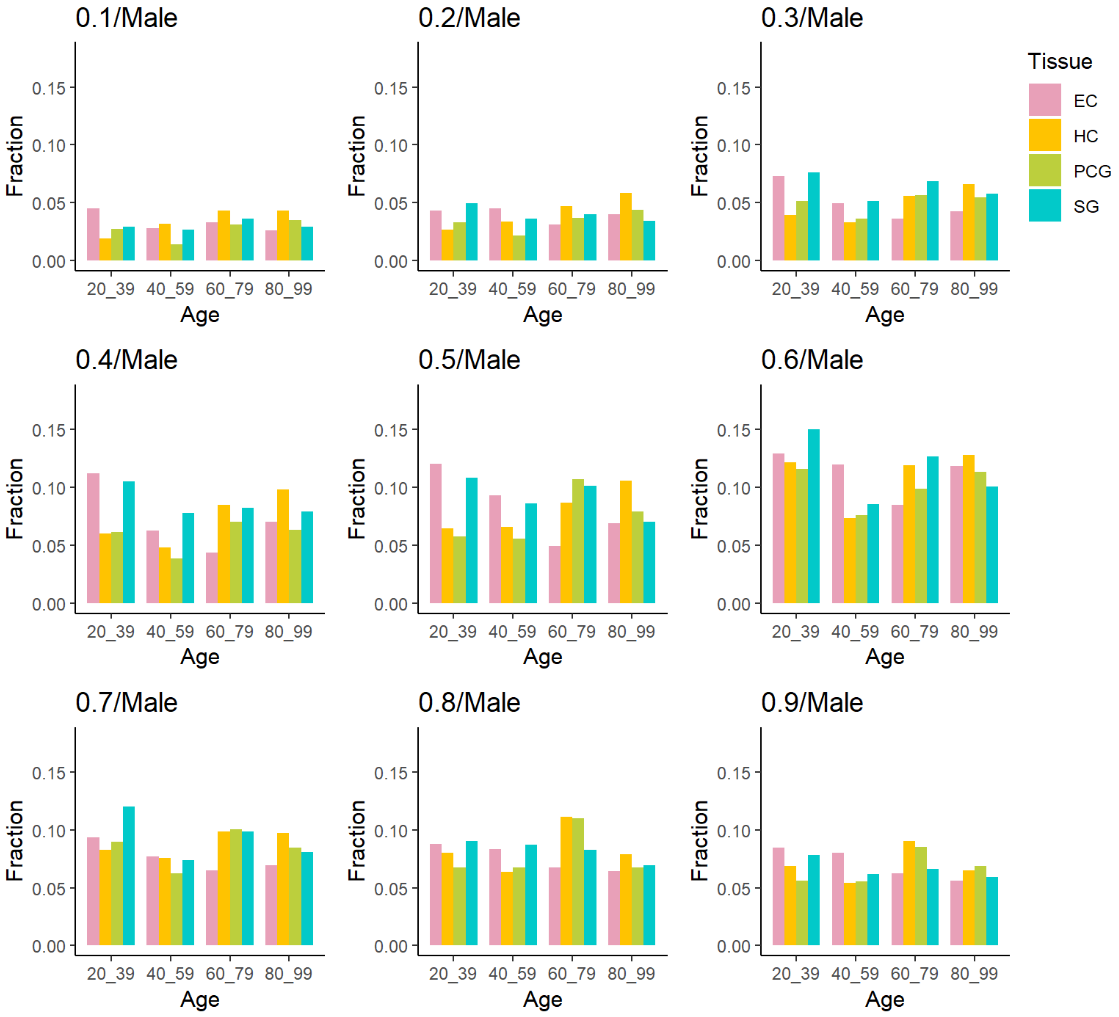

Fig. S23. The fraction of unique critical proteins at specific ages and each brain tissue for male samples

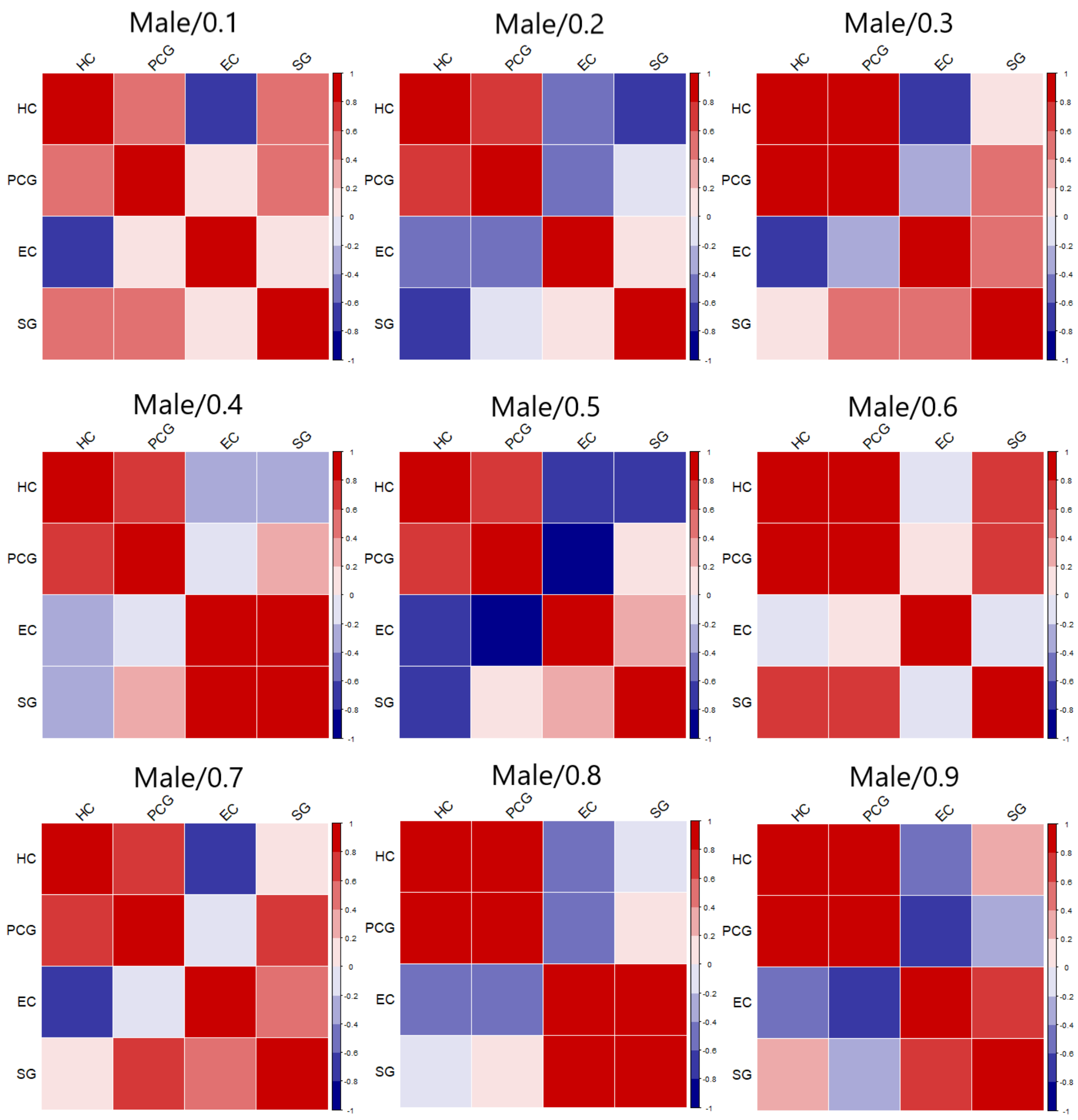

Fig. S24. The correlation coefficient matrix between brain regions computed from Fig. S23 for male samples. Red denotes positive correlation and blue indicates negative correlation.

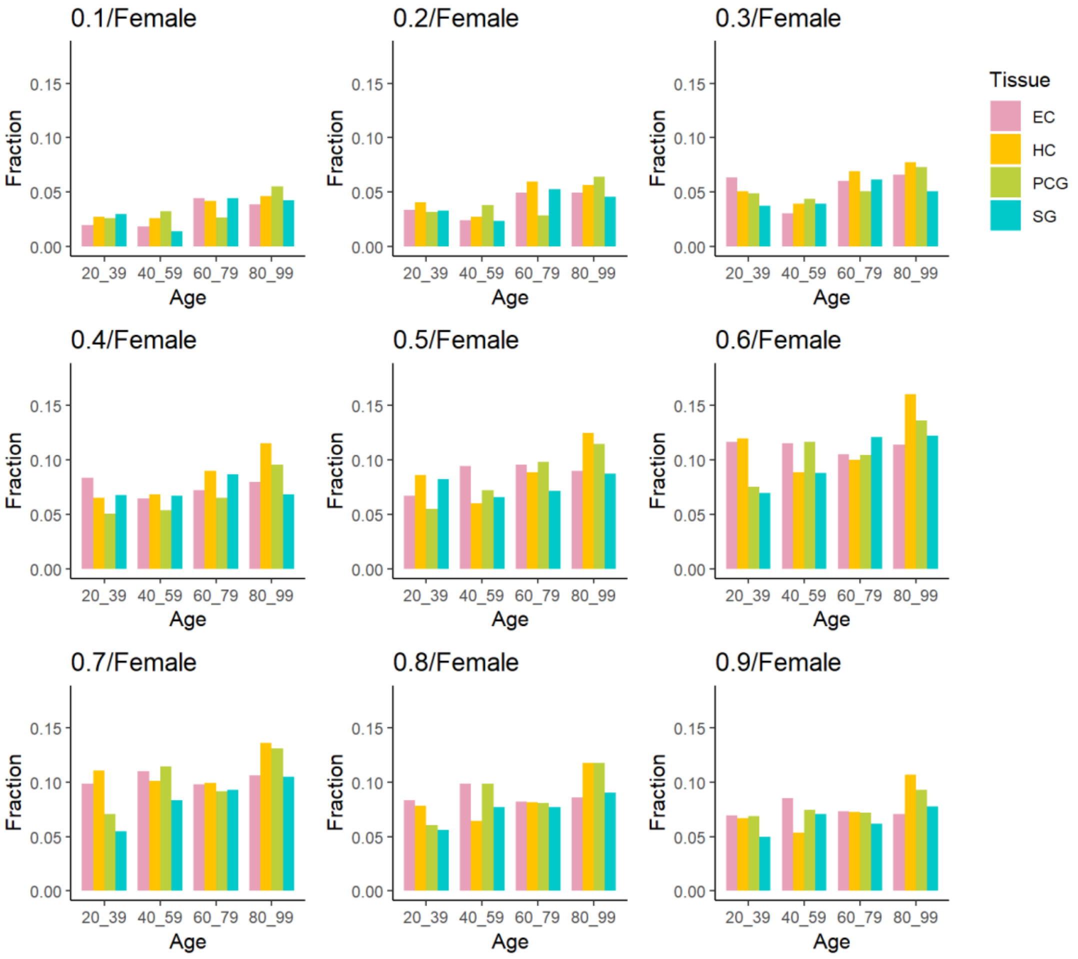

Fig. S25. The fraction of unique critical proteins at specific ages and each brain tissue for female samples

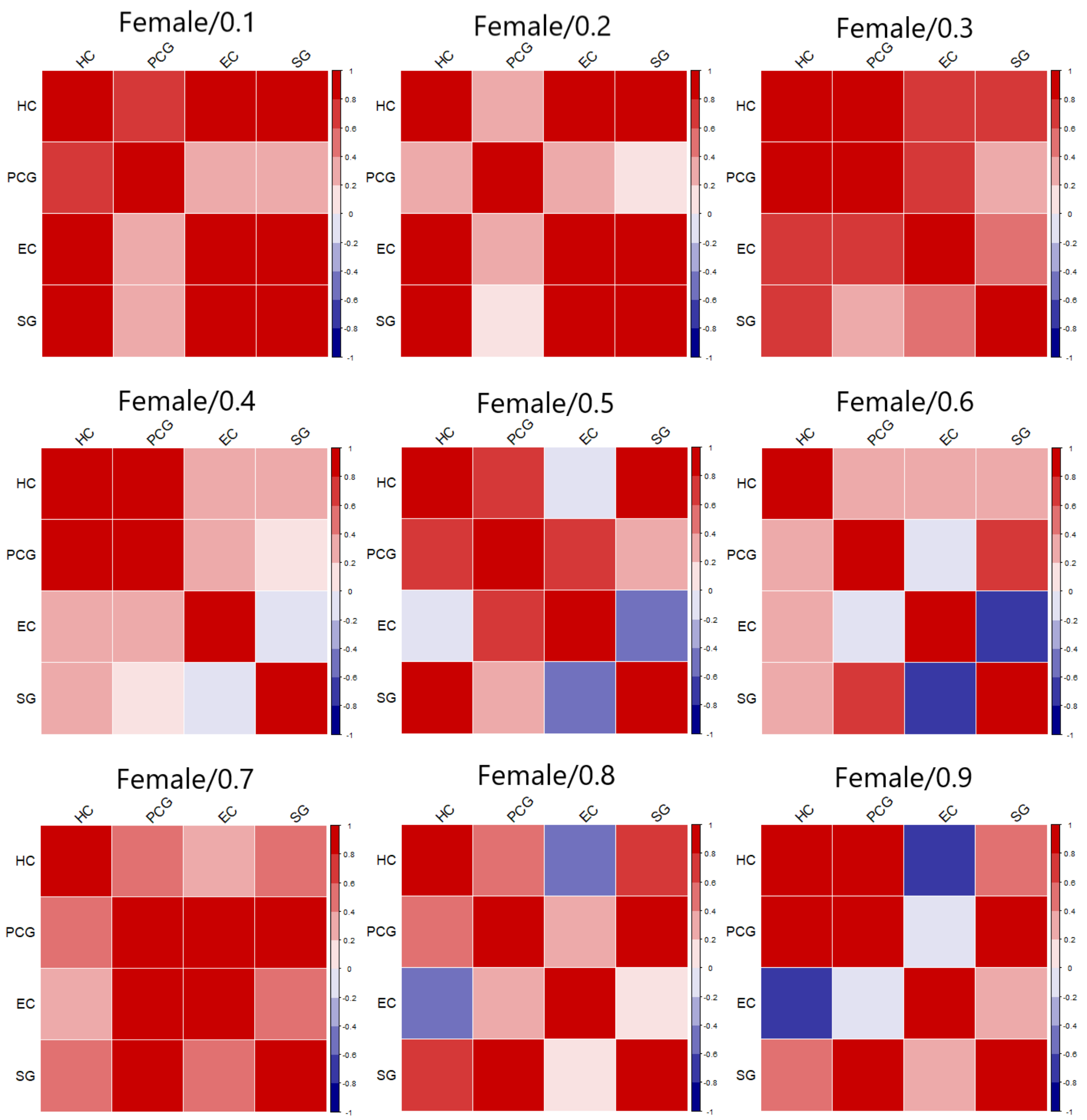

Fig. S26. The correlation coefficient matrix between brain regions computed from Fig. S25 for female samples. Red denotes positive correlation and blue indicates negative correlation.

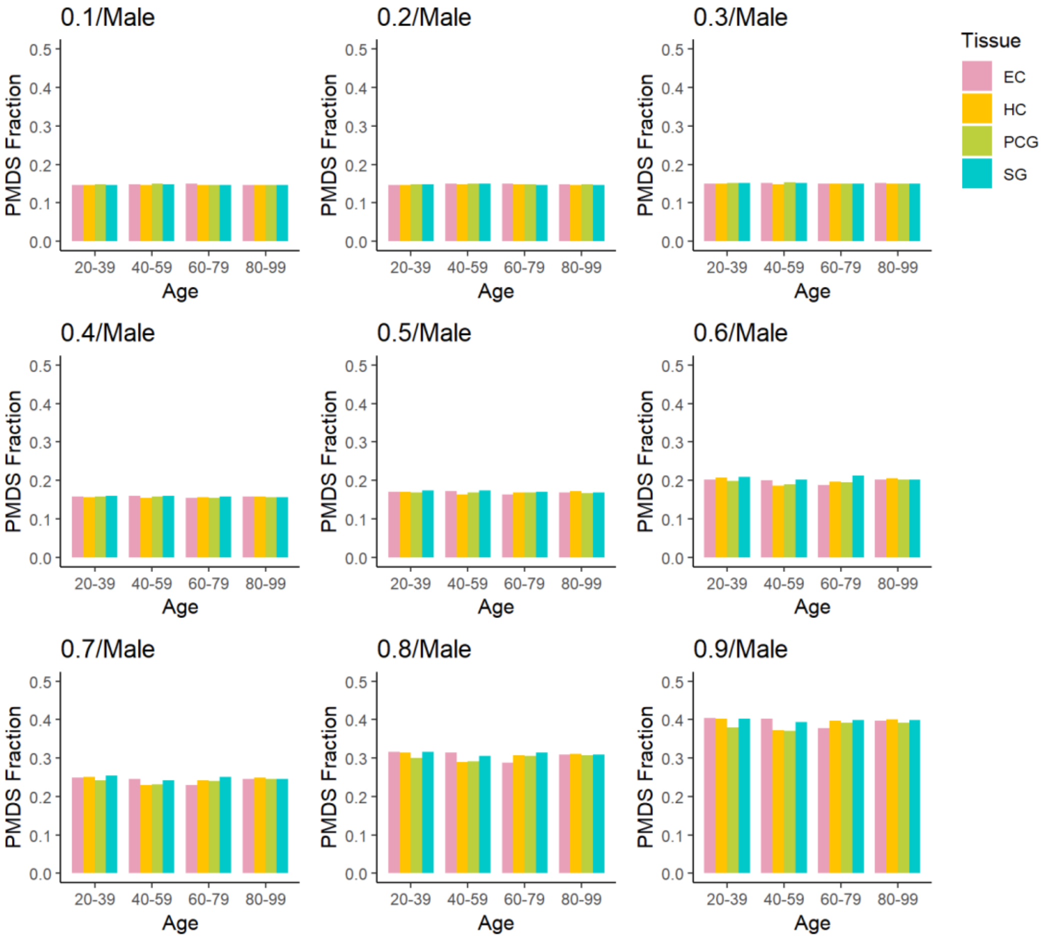

Fig. S27. The PMDS fraction of proteins at specific ages and each brain tissue for male samples

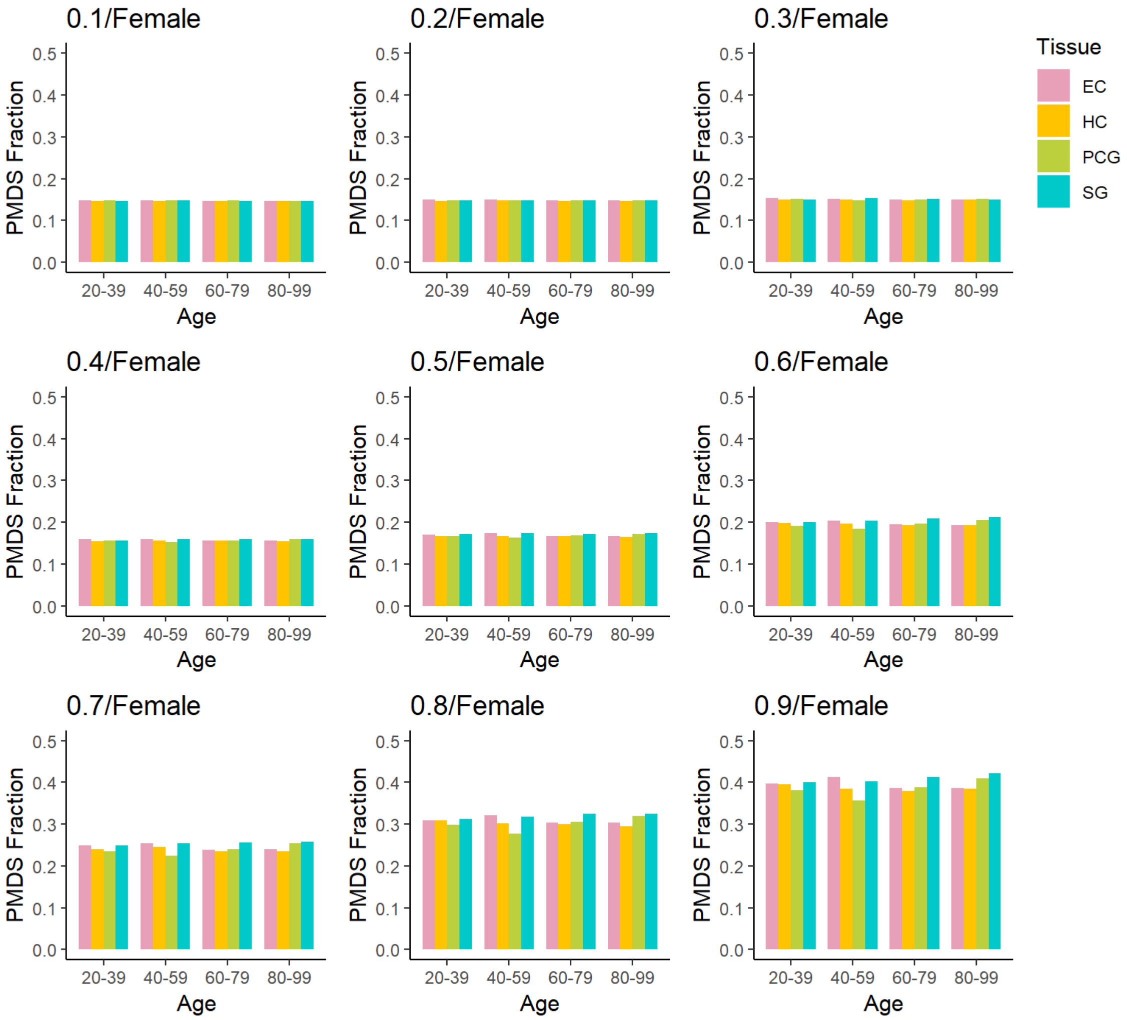

Fig. S28. The fraction of unique critical proteins at specific ages and each brain tissue for female samples

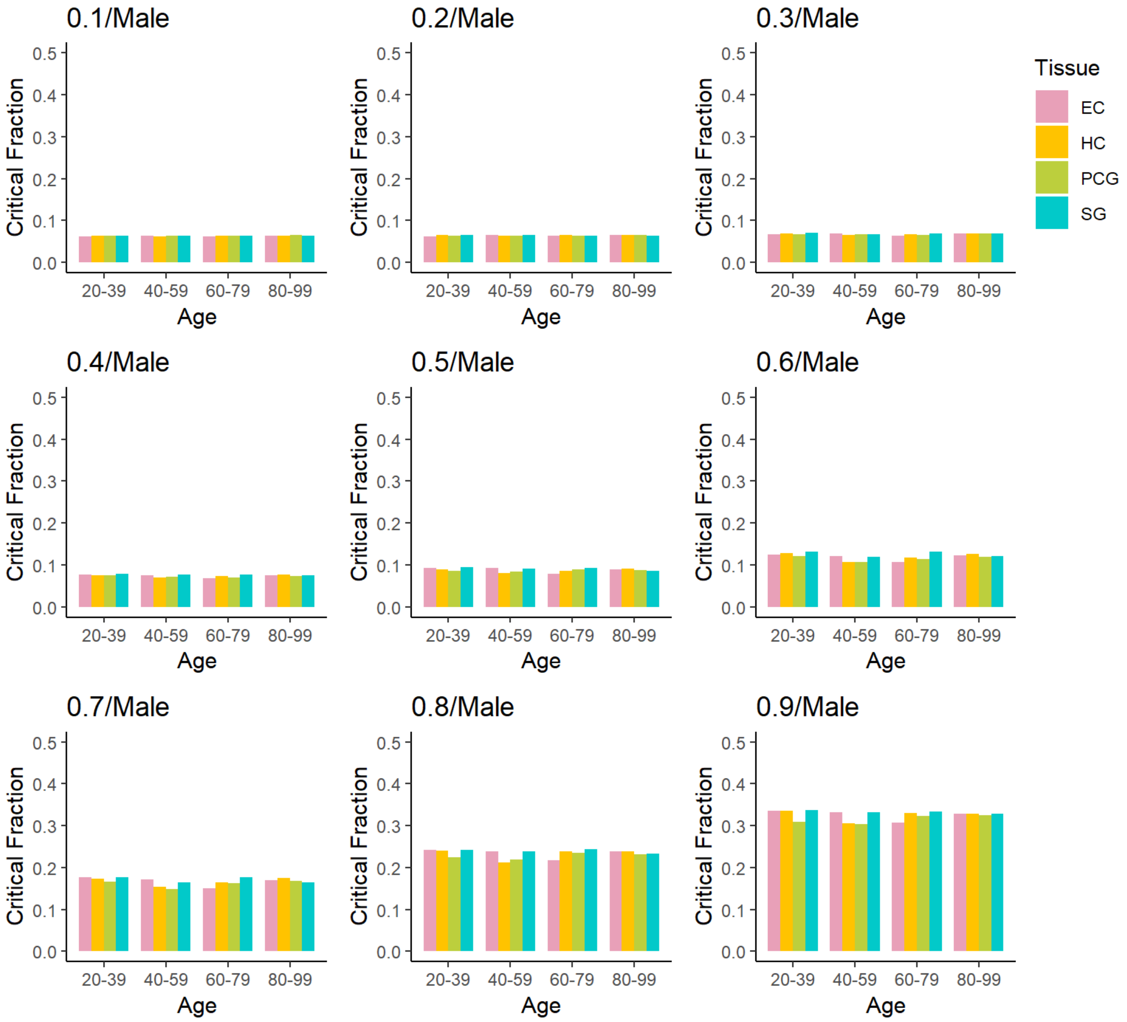

Fig. S29. The fraction of critical proteins at specific ages and each brain tissue for male samples

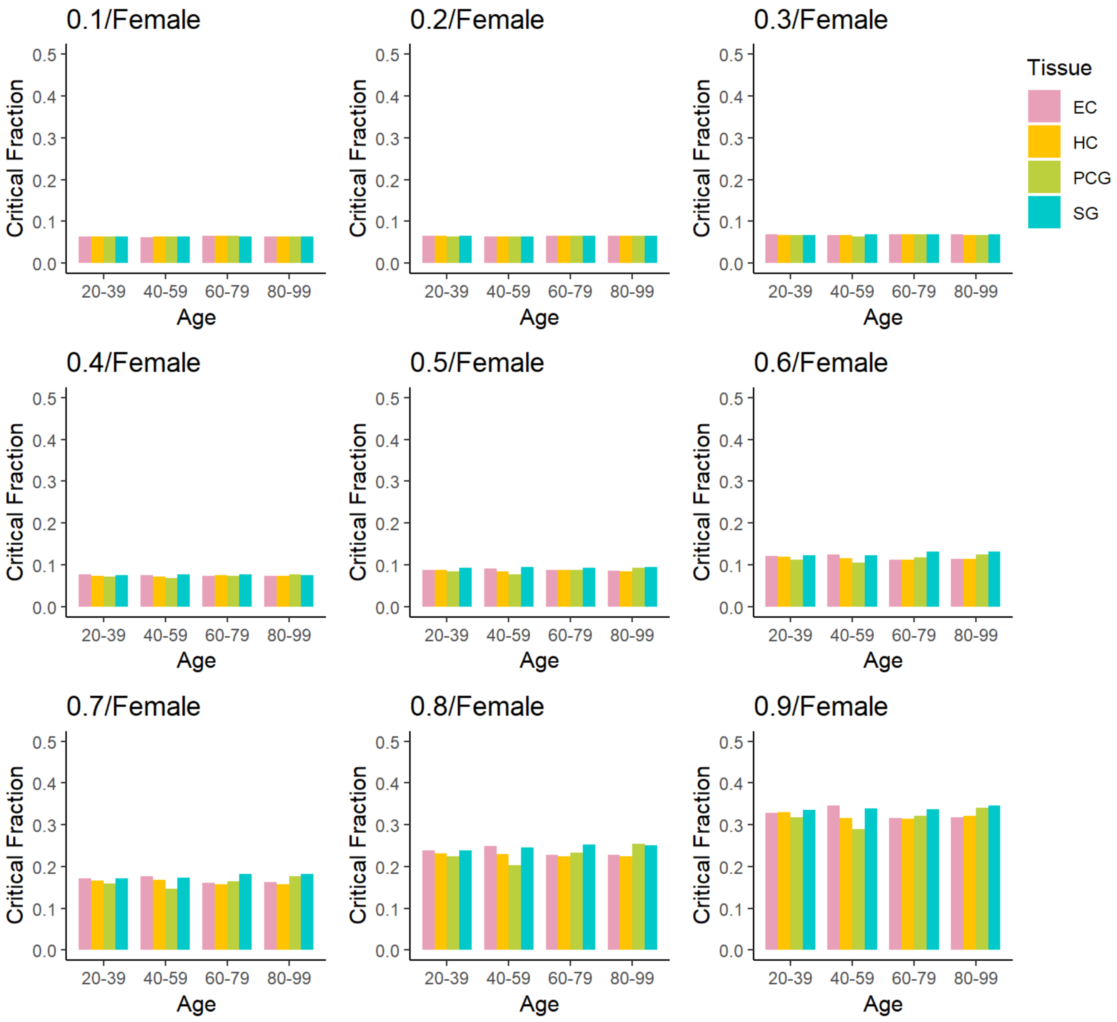

Fig. S30. The fraction of critical proteins at specific ages and each brain tissue for female samples

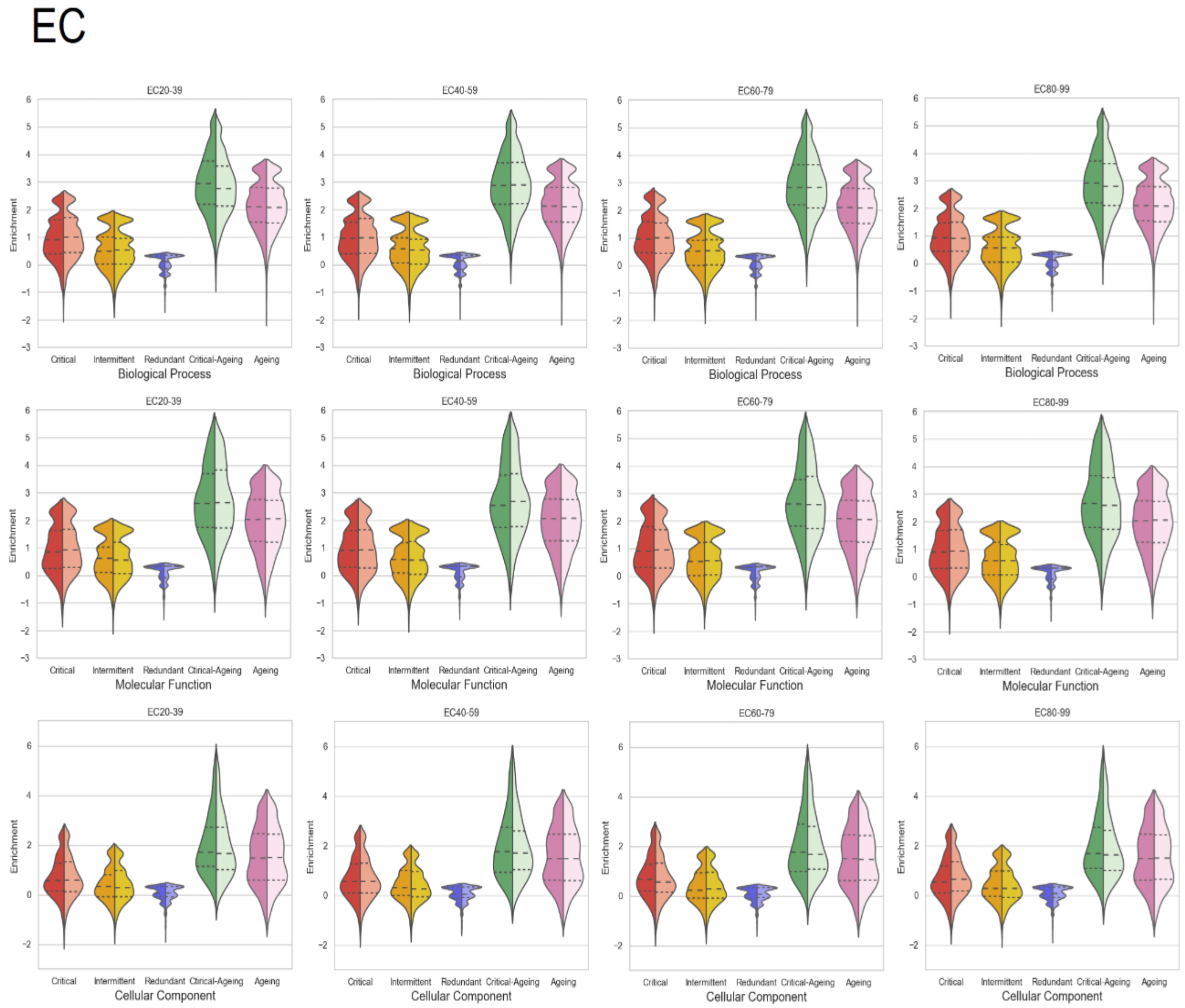

Fig. S31. Same as Fig. 9 for EC brain tissue.

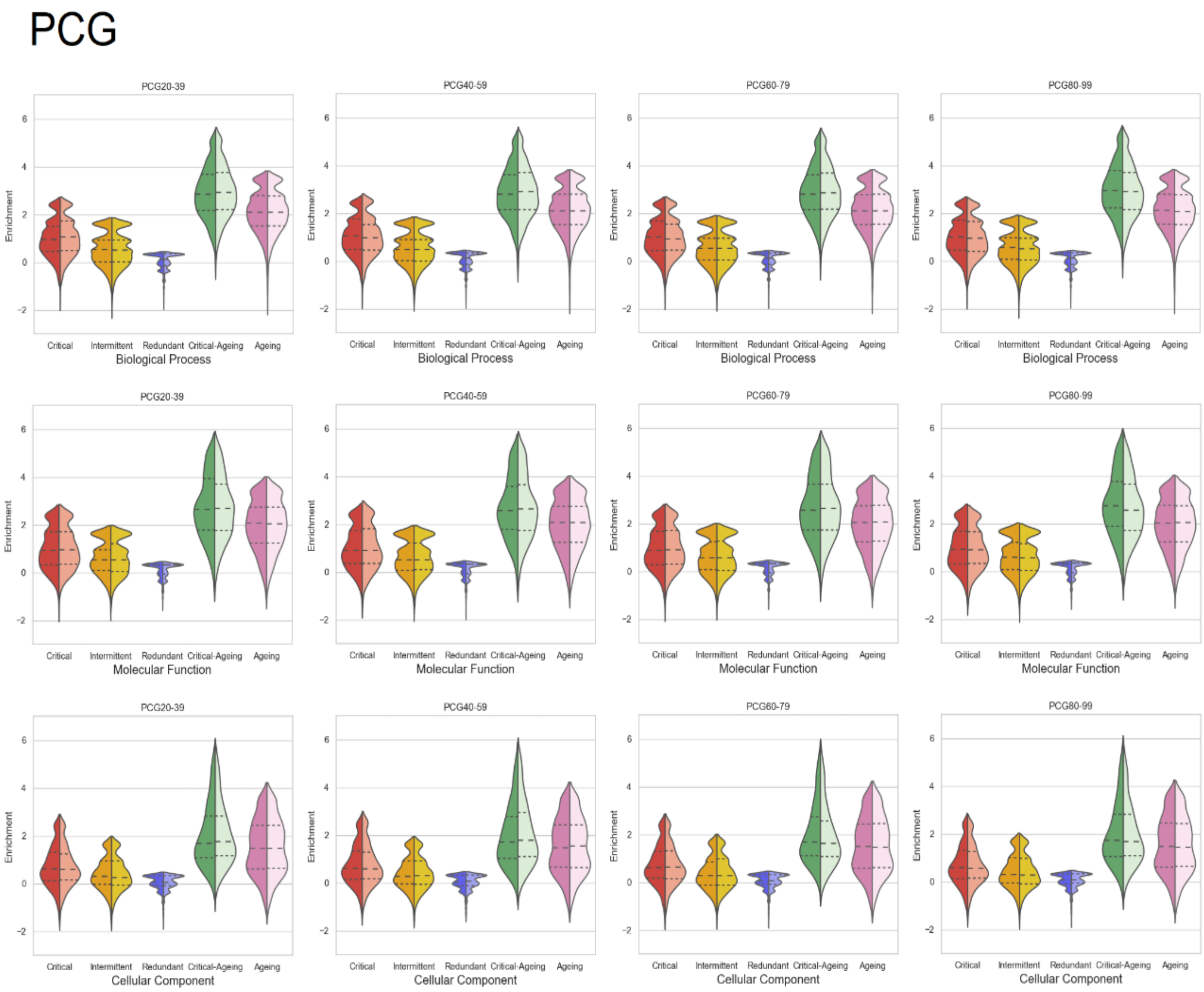

Fig. S32. Same as Fig. 9 for PCG brain tissue.

SG

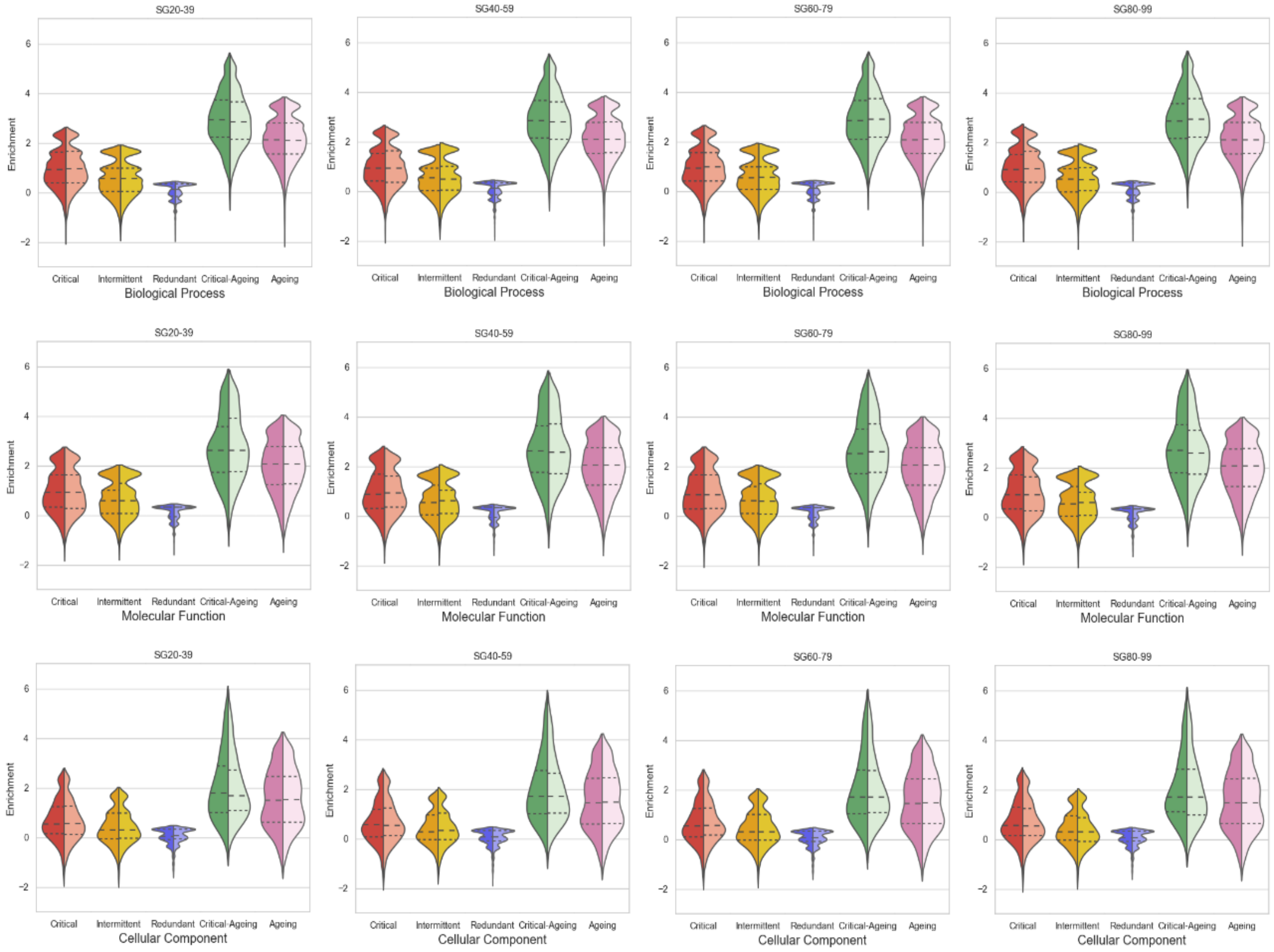

Fig. S33. Same as Fig. 9 for SG brain tissue.

Male/HC/0.5

replicative senescence(GO:0090399)

Age	p-Value	Enrichment (critical)	Critical Gene
20-39	0.012885	1.6831	ATM, CDKN1A, CTC1
40-59	0.010094	1.7705	ATM, CDKN1A, CTC1
60-79	0.011553	1.7222	ATM, CDKN1A, CTC1
80-99	0.132548	1.1223	ATM, CDKN1A

stress-induced premature senescence(GO:0090400)

Age	p-Value	Enrichment (critical)	Critical Gene
20-39	0.024259	1.9707	MAPK14, CDKN1A
40-59	0.000614	2.4636	MAPK14, CDKN1A, MAPKAPK5
60-79	0.022487	2.0099	MAPK14, CDKN1A
80-99	0.000801	2.3751	MAPK14, CDKN1A, MAPKAPK5

red : well-known aging gene

Table S1: Enrichment of critical proteins for specific aging biological processes such as replicative senescence and stress-induced premature senescense. Red indicates well-known aging genes according to the GenAge database. Black genes had not been associated before as aging genes.

Table S2 (Excel file): The P-values calculated using a two-tailed Fisher’s exact test associated to the enrichments shown in Figs 7 and S7.

#EC20-39 (θ = 0.5)	Male		Female	
Gene ontology (biological process)	P-value	Enrichment	P-value	Enrichment
Fc-gamma receptor signaling pathway involved in phagocytosis	1.12E-06	1.255088124	7.66E-06	1.222959453
histone mRNA catabolic process	5.19E-06	2.1808576	3.92E-06	2.228045683
positive regulation of transcription from RNA polymerase II prom	8.39E-06	2.33500828	6.62E-06	2.382196363
transforming growth factor beta receptor signaling pathway	9.18E-06	1.089792517	1.85E-05	1.082913379
cellular response to organic substance	1.55E-05	1.641861099	5.70E-06	1.746207597
negative regulation of protein catabolic process	2.41E-05	1.502099157	1.E-03	1.332374239
positive regulation of protein kinase B signaling	3.90E-05	0.915191227	2.44E-05	0.972371505
viral process	4.42E-05	0.585808425	3.60E-05	0.612415801
positive regulation of gene expression	5.08E-05	0.682085255	2.22E-05	0.733537738
intracellular signal transduction	6.93E-05	0.601745017	2.E-04	0.590436894

#EC40-59 (θ = 0.5)	Male		Female	
Gene ontology (biological process)	P-value	Enrichment	P-value	Enrichment
positive regulation of transcription, DNA-templated	2.75E-06	0.597928908	3.13E-05	0.544753173
histone mRNA catabolic process	5.20E-06	2.180275019	4.48E-06	2.205484379
positive regulation of transcription from RNA polymerase II prom	8.42E-06	2.334425699	7.42E-06	2.359635059
Fc-gamma receptor signaling pathway involved in phagocytosis	1.06E-05	1.194991416	1.54E-06	1.280825398
cellular response to hepatocyte growth factor stimulus	1.91E-05	2.046743626	4.55E-05	1.954169951
transforming growth factor beta receptor signaling pathway	3.55E-05	1.035142715	3.16E-05	1.045314198
cellular response to gamma radiation	4.48E-05	1.535918003	0.001681	1.309812935
hepatocyte growth factor receptor signaling pathway	4.64E-05	2.152104142	0.001013	1.954169951
nuclear mRNA surveillance	5.26E-05	1.928960591	4.55E-05	1.954169951
establishment of cell polarity	8.57E-05	1.378914254	0.0001	1.366383286

#EC60-79 (θ = 0.5)	Male		Female	
Gene ontology (biological process)	P-value	Enrichment	P-value	Enrichment
transforming growth factor beta receptor signaling pathway	8.64E-07	1.247369302	3.99E-06	1.146254907
viral process	1.28E-06	0.730239481	9.69E-05	0.585005053
histone mRNA catabolic process	2.03E-06	2.338434385	3.71E-06	2.23731999
positive regulation of transcription from RNA polymerase II prom	3.81E-06	2.492585065	6.32E-06	2.39147067
Fc-gamma receptor signaling pathway involved in phagocytosis	2.65E-05	1.239822096	3.13E-05	1.167695238
positive regulation of protein kinase B signaling	4.01E-05	1.006207245	1.10E-05	0.993876753
negative regulation of wound healing	4.63E-05	2.492585065	0.000896	1.986005562
autophagosome maturation	9.11E-05	1.730445013	0.001395	1.475179938
ubiquitin-dependent protein catabolic process	0.000101	0.705888898	0.000254	0.629564164
establishment of cell polarity	0.000147	1.431713104	0.000319	1.330598709

#EC80-99 (θ = 0.5)	Male		Female	
Gene ontology (biological process)	P-value	Enrichment	P-value	Enrichment
positive regulation of protein kinase B signaling	3.53E-06	1.030354324	2.75E-05	0.967283656
histone mRNA catabolic process	3.89E-06	2.229345798	3.30E-06	2.257246908
transforming growth factor beta receptor signaling pathway	4.49E-06	1.138280715	2.97E-06	1.166181825
positive regulation of transcription from RNA polymerase II prom	6.58E-06	2.383496478	5.72E-06	2.411397588
cellular response to hepatocyte growth factor stimulus	1.43E-05	2.095814405	0.000982	1.718250407
somitogenesis	3.02E-05	1.584988782	0.004987	1.258718078
hepatocyte growth factor receptor signaling pathway	3.65E-05	2.201174921	0.011682	1.718250407
negative regulation of protein catabolic process	7.56E-05	1.489678602	9.17E-05	1.473127949
positive regulation of gene expression	8.20E-05	0.689501177	0.000381	0.640691528
Fc-gamma receptor signaling pathway involved in phagocytosis	0.000113	1.110530802	2.47E-05	1.187622156

Table S3: Top 10 Gene Ontology (Biological Process) terms significantly enriched with critical proteins for EC brain tissue and each age range in males and females.

#HC20-39 (θ = 0.5)	Male		Female	
Gene ontology (biological process)	P-value	Enrichment	P-value	Enrichment
Fc-gamma receptor signaling pathway involved in phagocytosis	5.37E-08	1.388861868	2.16E-05	1.196613498
transforming growth factor beta receptor signaling pathway	1.96E-06	1.15247309	3.48E-06	1.155370539
histone mRNA catabolic process	4.06E-06	2.222097842	3.51E-06	2.246435622
positive regulation of transcription from RNA polymerase II prom	6.83E-06	2.376248522	6.04E-06	2.400586302
positive regulation of transcription by RNA polymerase II	1.89E-05	0.447491452	0.000159	0.414670818
cellular response to organic substance	1.92E-05	1.62903412	8.40E-05	1.589656086
positive regulation of protein kinase B signaling	2.96E-05	0.934691482	0.000228	0.879117162
thymus development	2.97E-05	1.406847965	0.002043	1.184190978
positive regulation of gene expression	7.18E-05	0.679323155	0.000141	0.67134719
ephrin receptor signaling pathway	7.82E-05	1.054492682	0.00062	0.962106188

#HC40-59 (θ = 0.5)	Male		Female	
Gene ontology (biological process)	P-value	Enrichment	P-value	Enrichment
histone mRNA catabolic process	2.42E-06	2.309493261	2.72E-06	2.289461912
positive regulation of transcription from RNA polymerase II prom	4.40E-06	2.463643941	4.87E-06	2.443612591
transforming growth factor beta receptor signaling pathway	6.00E-06	1.164360957	1.83E-06	1.198396828
Fc-gamma receptor signaling pathway involved in phagocytosis	2.19E-05	1.252553669	9.79E-06	1.259842494
positive regulation of gene expression	2.50E-05	0.753562502	4.51E-05	0.730634
positive regulation of protein kinase B signaling	2.50E-05	1.005028918	1.01E-06	1.101769993
establishment of cell polarity	2.81E-05	1.508132496	0.000881	1.304178308
T cell costimulation	4.00E-05	1.569826065	0.000329	1.43201168
negative regulation of protein binding	4.72E-05	1.190678265	0.000245	1.096538943
cellular response to organic substance	5.34E-05	1.652713724	3.71E-05	1.689840789

#HC60-79 (θ = 0.5)	Male		Female	
Gene ontology (biological process)	P-value	Enrichment	P-value	Enrichment
transforming growth factor beta receptor signaling pathway	6.17E-07	1.221451759	3.59E-06	1.153509386
histone mRNA catabolic process	3.22E-06	2.261223548	3.55E-06	2.244574469
positive regulation of transcription from RNA polymerase II prom	5.61E-06	2.415374228	6.10E-06	2.398725148
viral process	1.78E-05	0.638213558	0.000522	0.5305927
cellular response to hepatocyte growth factor stimulus	3.30E-05	2.00990912	3.63E-05	1.99326004
ubiquitin-dependent protein catabolic process	4.30E-05	0.695588258	0.000396	0.621780765
hepatocyte growth factor receptor signaling pathway	0.000101	2.078901991	0.00011	2.062252912
cell surface receptor signaling pathway	0.000124	0.985907695	0.004955	0.737327497
positive regulation of gene expression	0.000186	0.671623978	3.22E-05	0.719553898
positive regulation of protein kinase B signaling	0.000188	0.893905088	0.000125	0.894647752

#HC80-99 (θ = 0.5)	Male		Female	
Gene ontology (biological process)	P-value	Enrichment	P-value	Enrichment
positive regulation of gene expression	4.83E-07	0.815713822	1.26E-06	0.806054036
transforming growth factor beta receptor signaling pathway	2.00E-06	1.151365882	1.31E-05	1.111673809
histone mRNA catabolic process	4.09E-06	2.220990634	2.77E-06	2.286659076
signal transduction	6.37E-06	0.511689916	0.00536	0.352929346
positive regulation of transcription from RNA polymerase II prom	6.87E-06	2.375141314	4.94E-06	2.440809756
cellular response to organic substance	1.94E-05	1.627926912	0.000498	1.496348147
BMP signaling pathway	3.90E-05	1.251211217	0.001787	1.054515395
Fc-gamma receptor signaling pathway involved in phagocytosis	4.90E-05	1.131947796	0.000122	1.109575172
positive regulation of protein kinase B signaling	7.98E-05	0.897875175	0.002673	0.741423607
cellular response to hepatocyte growth factor stimulus	9.59E-05	1.86431569	0.007813	1.524519024

Table S4: Top 10 Gene Ontology (Biological Process) terms significantly enriched with critical proteins for HC brain tissue and each age range in males and females.

#PCG 20-39 ($\theta = 0.5$)	Male		Female	
Gene ontology (biological process)	P-value	Enrichment	P-value	Enrichment
histone mRNA catabolic process	3.36E-06	2.253965193	3.01E-06	2.272285343
positive regulation of transcription from RNA polymerase II prom	5.82E-06	2.408115873	5.31E-06	2.426436023
transforming growth factor beta receptor signaling pathway	1.30E-05	1.108832889	2.37E-06	1.18122026
negative regulation of protein catabolic process	1.98E-05	1.532647135	8.15E-05	1.488166384
Fc-gamma receptor signaling pathway involved in phagocytosis	2.57E-05	1.184340441	3.23E-06	1.28700174
negative regulation of protein binding	3.32E-05	1.164922355	7.11E-05	1.153470347
cellular protein-containing complex localization	3.44E-05	2.002650765	0.005299	1.615505807
nuclear mRNA surveillance	3.44E-05	2.002650765	0.000494	1.838649358
establishment of cell polarity	4.55E-05	1.452604428	3.88E-05	1.470924578
positive regulation of transcription by RNA polymerase II	6.24E-05	0.437204422	3.39E-05	0.456995376

#PCG 40-59 ($\theta = 0.5$)	Male		Female	
Gene ontology (biological process)	P-value	Enrichment	P-value	Enrichment
histone mRNA catabolic process	2.88E-06	2.279604148	1.86E-06	2.353202789
cellular response to organic substance	3.71E-06	1.797766061	0.000217	1.620050274
Fc-gamma receptor signaling pathway involved in phagocytosis	3.93E-06	1.274517918	1.73E-05	1.275209787
positive regulation of transcription from RNA polymerase II prom	5.11E-06	2.433754828	3.53E-06	2.507353469
negative regulation of protein binding	8.32E-06	1.270604018	0.000103	1.180482528
cellular protein-containing complex localization	2.97E-05	2.02828972	0.000337	1.919566804
nuclear mRNA surveillance	2.97E-05	2.02828972	0.000337	1.919566804
transforming growth factor beta receptor signaling pathway	3.64E-05	1.07731343	3.22E-06	1.208070485
positive regulation of transcription, DNA-templated	3.87E-05	0.561952651	4.74E-05	0.572937699
intracellular signal transduction	3.95E-05	0.654265451	0.000225	0.627040602

#PCG 60-79 ($\theta = 0.5$)	Male		Female	
Gene ontology (biological process)	P-value	Enrichment	P-value	Enrichment
Fc-gamma receptor signaling pathway involved in phagocytosis	1.17E-06	1.3008757	4.91E-07	1.355880662
histone mRNA catabolic process	3.98E-06	2.225534682	3.66E-06	2.239717649
transforming growth factor beta receptor signaling pathway	4.76E-06	1.134469599	8.73E-07	1.19994586
positive regulation of transcription from RNA polymerase II prom	6.71E-06	2.379685361	6.25E-06	2.393868329
cellular response to organic substance	1.07E-05	1.686538181	5.18E-06	1.757879562
positive regulation of protein kinase B signaling	1.61E-05	0.970918144	2.05E-05	0.98404347
positive regulation of cell migration	2.59E-05	0.84720849	0.000738	0.717178189
viral process	4.26E-05	0.602524692	1.46E-05	0.634544135
intracellular signal transduction	4.60E-05	0.627456731	0.000625	0.552098439
positive regulation of gene expression	5.82E-05	0.693286408	0.000105	0.673204781

#PCG 80-99 ($\theta = 0.5$)	Male		Female	
Gene ontology (biological process)	P-value	Enrichment	P-value	Enrichment
transforming growth factor beta receptor signaling pathway	9.04E-07	1.197668938	1.88E-06	1.151689084
histone mRNA catabolic process	3.71E-06	2.237440727	4.87E-06	2.191460873
positive regulation of gene expression	3.71E-06	0.782153494	0.00061	0.600372099
positive regulation of transcription from RNA polymerase II prom	6.32E-06	2.391591407	7.96E-06	2.345611552
cellular response to organic substance	9.70E-06	1.698444226	0.000592	1.458308357
intracellular signal transduction	2.30E-05	0.651533312	0.000264	0.561820253
nuclear mRNA surveillance	3.78E-05	1.986126298	0.000722	1.757824888
Fc-gamma receptor signaling pathway involved in phagocytosis	4.03E-05	1.148397889	1.23E-05	1.186374642
establishment of cell polarity	5.25E-05	1.436079962	7.80E-05	1.390100107
cellular response to hepatocyte growth factor stimulus	8.73E-05	1.880765783	0.000229	1.739475749

Table S5: Top 10 Gene Ontology (Biological Process) terms significantly enriched with critical proteins for PCG brain tissue and each age range in males and females.

#SG 20-39 (θ = 0.5)	Male		Female	
Gene ontology (biological process)	P-value	Enrichment	P-value	Enrichment
transforming growth factor beta receptor signaling pathway	2.88E-06	1.124188695	9.82E-06	1.084894004
histone mRNA catabolic process	5.73E-06	2.163960484	5.34E-06	2.175959087
positive regulation of transcription from RNA polymerase II prom	9.13E-06	2.318111164	8.60E-06	2.330109766
Fc-gamma receptor signaling pathway involved in phagocytosis	1.31E-05	1.17867688	3.82E-05	1.146339669
cellular response to gamma radiation	5.11E-05	1.519603467	0.001999	1.280287642
cellular protein-containing complex localization	5.78E-05	1.912646055	0.000775	1.742323101
nuclear mRNA surveillance	5.78E-05	1.912646055	5.39E-05	1.924644658
cellular response to hepatocyte growth factor stimulus	5.78E-05	1.912646055	5.39E-05	1.924644658
cell surface receptor signaling pathway	8.27E-05	0.945062029	0.00238	0.785210375
cellular response to organic substance	9.20E-05	1.564339361	8.44E-05	1.576337964

#SG 40-59 (θ = 0.5)	Male		Female	
Gene ontology (biological process)	P-value	Enrichment	P-value	Enrichment
histone mRNA catabolic process	4.51E-06	2.204378415	6.17E-06	2.151387794
positive regulation of transcription from RNA polymerase II prom	7.46E-06	2.358529095	9.73E-06	2.305538474
transforming growth factor beta receptor signaling pathway	8.24E-06	1.098275454	1.08E-06	1.145368292
positive regulation of protein kinase B signaling	1.02E-05	0.995224252	1.05E-06	1.035777929
positive regulation of transcription, DNA-templated	1.53E-05	0.569697885	6.07E-06	0.569041683
negative regulation of protein binding	2.72E-05	1.174758998	2.04E-05	1.146301563
nuclear mRNA surveillance	4.58E-05	1.953063986	6.21E-05	1.900073366
cellular response to hepatocyte growth factor stimulus	4.58E-05	1.953063986	6.21E-05	1.900073366
cellular response to organic substance	6.87E-05	1.604757292	0.000101	1.551766672
establishment of cell polarity	6.96E-05	1.403017649	0.00011	1.350027029

#SG 60-79 (θ = 0.5)	Male		Female	
Gene ontology (biological process)	P-value	Enrichment	P-value	Enrichment
transforming growth factor beta receptor signaling pathway	2.70E-06	1.130110236	2.83E-06	1.128777478
histone mRNA catabolic process	5.06E-06	2.184919902	4.67E-06	2.19840223
positive regulation of transcription from RNA polymerase II prom	8.22E-06	2.339070582	7.69E-06	2.35255291
negative regulation of protein binding	4.49E-05	1.135097778	0.0002	1.059784607
positive regulation of reactive oxygen species metabolic process	4.57E-05	1.645923402	0.001711	1.436262178
Fc-gamma receptor signaling pathway involved in phagocytosis	5.80E-05	1.115295151	6.30E-06	1.233321334
positive regulation of transcription, DNA-templated	6.46E-05	0.521777811	2.12E-05	0.544264138
establishment of cell polarity	0.00012	1.345818809	0.000107	1.359301137
cellular response to organic substance	0.00013	1.528140366	1.34E-05	1.659405729
negative regulation of telomere maintenance via telomerase	0.00013	1.528140366	0.003586	1.311099035

#SG 80-99 (θ = 0.5)	Male		Female	
Gene ontology (biological process)	P-value	Enrichment	P-value	Enrichment
Fc-gamma receptor signaling pathway involved in phagocytosis	1.37E-06	1.294902776	2.25E-05	1.141014954
histone mRNA catabolic process	3.27E-06	2.25878247	5.68E-06	2.165519271
transforming growth factor beta receptor signaling pathway	3.72E-06	1.15267951	5.40E-05	1.005349089
positive regulation of transcription from RNA polymerase II prom	5.68E-06	2.41293315	9.06E-06	2.319669951
viral process	3.23E-05	0.624803449	0.001236	0.487088487
T cell costimulation	5.99E-05	1.519115274	0.000187	1.381400312
positive regulation of transcription by RNA polymerase II	6.70E-05	0.431495286	0.001095	0.350229304
cellular response to organic substance	7.69E-05	1.602002934	9.10E-05	1.565898148
negative regulation of protein binding	8.25E-05	1.139967474	0.000534	1.013418297
SMAD protein signal transduction	9.07E-05	1.474663512	0.000766	1.308069039

Table S6: Top 10 Gene Ontology (Biological Process) terms significantly enriched with critical proteins for SG brain tissue and each age range in males and females.

Tables S7-S18 include the separate results for the enrichment of each Gene Ontology (Biological process, Cellular component and Molecular function) term with critical proteins for each brain tissue (SG, PCG, HC, EC) and each age range in males and females. The EXCEL files can be downloaded electronically.

TableS7_BiologicalProcess_SG
TableS8_BiologicalProcess_PCG
TableS9_BiologicalProcess_HC
TableS10_BiologicalProcess_EC
TableS11_CellularComponent_SG
TableS12_CellularComponent_PCG
TableS13_CellularComponent_HC
TableS14_CellularComponent_EC
TableS15_MolecularFunction_SG
TableS16_MolecularFunction_PCG
TableS17_MolecularFunction_HC
TableS18_MolecularFunction_EC
