# Supplementary material for: Probabilistic Critical Controllability Analysis of Protein Interaction Networks Integrating Normal Brain Ageing Gene Expression Profiles
Source: Int J Mol Sci. 2021 Sep 13;22(18):9891. doi: 10.3390/ijms22189891 (PMC8465977; doi:10.3390/ijms22189891)
Supplement: Supplementary file 1 [file ijms-22-09891-s001.zip › Code_CPMDS.zip!/Code_CPMDS/Instructions.docx]

How to run the program for the CPMDS (Critical probabilistic Minimum Dominating Set) :

First, the users need to set up a Java development environment and install IBM ILOG CPLEX Optimization (<https://www.ibm.com/products/ilog-cplex-optimization-studio>). The version CPLEX 12.8.0.0 was used in this work.

If you use an integrated development environment (Eclipse), you can import and run the program. To run it at the command prompt, please follow the steps below.

1. Go to the following folder: CPMDS_CriticalControllabilityAnalyzer/src
2. Execute the following commands:

>javac -encoding UTF-8 Main.java

>java Main

3. The program will request for the input folder, result folder as well as parameter θ as follows:

<example>

Input folder path (absolute path)	> C:\Users\xxx\xxx\Example\input

Result folder path (absolute path)	> C:\Users\ xxx\xxx \Example\result

Parameter (0 < θ < 1) > 0.5

As shown in the input folder, the input file only consists of an edge list of the network followed by a failure probability value as follows:

<example>

1 2 0.5

1 3 0.2

2 4 0.8

・

・

・
